# The Rice Dynamin-Related Protein OsDRP1E Negatively Regulates Programmed Cell Death by Controlling the Release of Cytochrome *c* from Mitochondria

**DOI:** 10.1371/journal.ppat.1006157

**Published:** 2017-01-12

**Authors:** Zhiqiang Li, Bo Ding, Xueping Zhou, Guo-Liang Wang

**Affiliations:** 1 State Key Laboratory for Biology of Plant Diseases and Insect Pests, Institute of Plant Protection, Chinese Academy of Agricultural Sciences, Beijing, China; 2 Southern Regional Collaborative Innovation Center for Grain and Oil Crops in China and College of Agronomy, Hunan Agricultural University, Changsha, Hunan, China; 3 Department of Plant Pathology, Ohio State University, Columbus, Ohio, United States of America; Nanjing Agricultural University, CHINA

## Abstract

Programmed cell death (PCD) mediated by mitochondrial processes has emerged as an important mechanism for plant development and responses to abiotic and biotic stresses. However, the role of translocation of cytochrome *c* from the mitochondria to the cytosol during PCD remains unclear. Here, we demonstrate that the rice dynamin-related protein 1E (OsDRP1E) negatively regulates PCD by controlling mitochondrial structure and cytochrome *c* release. We used a map-based cloning strategy to isolate *OsDRP1E* from the lesion mimic mutant *dj-lm* and confirmed that the E409V mutation in OsDRP1E causes spontaneous cell death in rice. Pathogen inoculation showed that *dj-lm* significantly enhances resistance to fungal and bacterial pathogens. Functional analysis of the E409V mutation showed that the mutant protein impairs OsDRP1E self-association and formation of a higher-order complex; this in turn reduces the GTPase activity of OsDRP1E. Furthermore, confocal microscopy showed that the E409V mutation impairs localization of OsDRP1E to the mitochondria. The E409V mutation significantly affects the morphogenesis of cristae in mitochondria and causes the abnormal release of cytochrome *c* from mitochondria into cytoplasm. Taken together, our results demonstrate that the mitochondria-localized protein OsDRP1E functions as a negative regulator of cytochrome *c* release and PCD in plants.

## Author Summary

Plants have developed a hypersensitive response (HR) that shows rapid programed cell death (PCD) around the infection site, which in turn limits pathogen invasion and restricts the spread of pathogens. Although many studies reported the characterization of PCD in different pathosystems in the last decade, the molecular mechanisms on how PCD is initiated and how it regulates host resistance are still unclear. Lesion mimic mutants exhibit spontaneous HR-like cell death without pathogen invasion and are ideal genetic materials for dissecting the PCD pathway. In this study, we characterized the lesion mimic gene *OsDRP1E* that negatively regulates plant PCD through the control of cytochrome *c* release from mitochondria. Our results suggest that the E409V point mutation in the dynamin-related protein OsDRP1E affects the morphogenesis of mitochondrial cristae that leads to the cytochrome *c* release into cytoplasm. This study provides new insights into the function of dynamin-related proteins in plant immunity.

## Introduction

Programmed cell death (PCD) occurs in animals and plants, and the primary forms of PCD in mammals include apoptosis, autophagy, and necrosis [1]. In plants, PCD occurs during normal growth, development, and responses to biotic and abiotic stresses [2]. In plant disease resistance, PCD in the hypersensitive reaction (HR) is accompanied by the accumulation of reactive oxygen species (ROS) [3] and is triggered by the activation of plant resistance proteins after recognition of their corresponding effectors from the pathogen. The HR characteristically involves spontaneous PCD around the infection sites, which limits pathogen invasion and restricts the spread of pathogens [4].

Some mutant plants exhibit spontaneous HR-like cell death even without pathogen invasion. Based on their cell death phenotypes, these mutants were designated lesion mimic or spotted leaf mutants [5]. A number of lesion mimic and spotted leaf mutants have been described in many plant species, including maize [6], *Arabidopsis thaliana* [7], barley [8], and rice [9,10]. To date, more than 60 lesion mimic genes have been identified in plants [5]. These genes encode proteins that play various regulatory roles in different pathways, such as sphingolipid and fatty acid biosynthesis [11], chloroplast activity and photosynthesis [12], transcriptional regulation [13], signal perception at the plasma membrane [14], Ca^2+^ signal transduction [15], and ubiquitination-mediated protein degradation [10]. Therefore, various pathways regulate the complex process of PCD in plants.

The mitochondrion, the bioenergy hub of the cell, plays central roles in biochemical pathways for energy production, signal transduction, and cellular metabolism [16,17]. In addition, mitochondria play a major role in the regulation of apoptosis in animals [18]. Cytochrome *c* plays an important part in this process, serving as one of the first markers of the molecular events preceding apoptosis [19]. During apoptosis, cytochrome *c*, the sole water-soluble component of the electron transfer chain, is released from the intermembrane space of the mitochondria into the cytosol [20,21]. Cytosolic cytochrome *c* binds to Apaf-1 to promote the assembly of apoptosomes and recruits procaspase-9 to these complexes, which subsequently initiates an apoptotic protease cascade [22]. Several proteins, including BH3, Bim, and tBid, are involved in the conformational changes to PCD-related proteins such as Bax and Bak, allowing them to form oligomers on the mitochondria. Oligomerized Bax and Bak trigger apoptosis by causing permeabilization of the mitochondrial outer membrane and activation of OMA1 [17].

Several lines of evidence demonstrated that mitochondria also participate in plant PCD [23,24]. The induction of PCD in *Arabidopsis* cell cultures by ceramide, protoporphyrin IX, or the avirulence factor AvrRpt2 leads to morphological changes in the mitochondria, as well as the release of cytochrome *c* [25]. A work in *Arabidopsis* also detected changes in the dynamics and morphology of mitochondria during the onset of cell death [26]. These findings suggest that mitochondria play a role in regulating PCD in plants, but it is still not clear how mitochondrial proteins regulate plant PCD [27]. In recent years, several studies have observed the release of cytochrome *c* from the mitochondria into the cytosol before plant cell death following toxin protein and elicitor treatments [28–30]. However, the proteins that regulate cytochrome *c* release during plant PCD are currently unknown.

Dynamin-related proteins (DRPs) and dynamin-like proteins belong to the structurally conserved yet functionally divergent dynamin superfamily. These proteins are commonly found in prokaryotic and eukaryotic organisms including mammals, plants, fungi and bacteria [31,32]. In general, classical dynamin family proteins have five distinct domains: the N-terminal GTPase domain, which binds to guanosine triphosphate (GTP) and hydrolyzes GTP to guanosine diphosphate; the “middle” domain, which is involved in the formation of homo-polymers based on self-interaction; the pleckstrin homology domain, which is related to lipid binding; the GTPase-effector domain, which interacts with the GTPase domain and regulates GTPase activity; and the C-terminal proline-rich domain, which participates in protein–dynamin interactions [31,33,34]. The multi-domain DRPs self-assemble into complex higher-order rings and helices and trigger the fusion or fission of organelles. Studies in *Arabidopsis* have shown that DRPs play various roles in different pathways: clathrin-dependent endocytosis (DRP1 subfamily) [35], induction of cell death (AtDRP1E during powdery mildew infection) [36], pinching of the clathrin-coated vesicles (DRP2A) [37], vesicular trafficking through the perception of PAMP-triggered immunity (PTI) signaling (DRP2B) [38], and regulated fission of mitochondria and peroxisomes (DRP3) [39] and chloroplasts (DRP5) [40]. However, the roles of rice DRPs remain poorly understood. To date, OsDRP2B is the only DRP that has been shown to regulate cellulose biosynthesis in rice [41,42].

In this study, we characterized a spontaneous lesion mimic mutant, designated *dj-lm* (*dongjin-lesion mimic*), which was found among plants of the japonica rice (*Oryza sativa*) cultivar Dongjin (DJ) grown in our greenhouse. Using map-based cloning, we cloned the mutated gene and found that the cell death phenotype of *dj-lm* resulted from a point mutation of the rice dynamin-related gene *OsDRP1E*. This point mutation abolished the self-interaction of OsDRP1Es in yeast, disrupted high-order complex formation *in planta* and reduced the protein’s intrinsic GTPase activity *in vitro*. Our results show that the E409 residue is required for the localization of OsDRP1E to the mitochondria, and the point mutation affects the morphology of the mitochondrial cristae and the release of cytochrome *c* into the cytoplasm, which leads to PCD in rice plants.

## Results

### Phenotypic characterization of the *dj-lm* mutant

**Fig 1:**
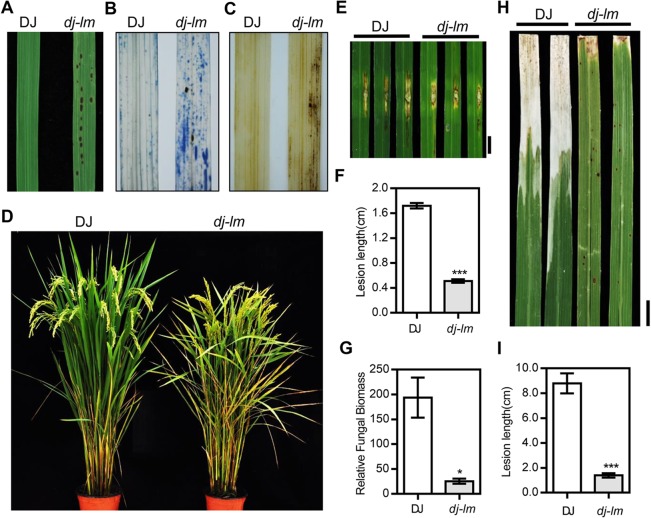
Phenotypic characterization of the *dj-lm* mutant. **(A)** Representative leaves of Dongjin (DJ) and *dj-lm* plants. **(B)** Trypan blue staining of DJ and *dj-lm* leaves. **(C)** Diamiobenzidine (DAB) staining of DJ and *dj-lm* leaves. **(D)** DJ and *dj-lm* plants grown in the field. **(E)** Disease phenotypes of DJ and *dj-lm* after inoculation with *M*. *oryzae* isolate RO1-1. Similar results were obtained from three independent experiments. Bar = 1 cm. **(F)** Lesion length of DJ and *dj-lm* after inoculation with RO1-1. Values are means ± standard errors of 10 replications. Significance was determined at ***P<0.0001 with a Student’s *t*-test. **(G)** Relative fungal biomass of DJ and *dj-lm* after inoculation with *M*. *oryzae*. Values are means ± standard errors of 10 replications. Significance was determined at *P<0.05 with a Student’s *t*-test. **(H)** Disease phenotypes of DJ and *dj-lm* after inoculation with *Xoo* strain PXO-99. Similar results were obtained from three independent experiments. Bar = 1 cm. **(I)** Lesion length of DJ and *dj-lm* after inoculation with PXO99. Values are means ± standard errors of 10 replications. Significance was determined at ***P<0.0001 with a Student’s *t*-test.

Under green house or field conditions, the leaves of *dj-lm* mutants showed small, dark brown lesions by 30 d to 45 d after germination (Fig 1A). The lesions increased in both quantity and size with the maximum abundance reached at around 2.5 month. Then the cell death lesions gradually covered the entire leaf area, aggravating from the tip to the whole leaf (S1 Fig). After Trypan blue staining, the *dj-lm* leaves exhibited numerous dark blue spots (Fig 1B), indicating the occurrence of extensive cell death. When we analyzed H_2_O_2_ accumulation using 3,3′-diaminobenzidine (DAB) staining, many brownish spots appeared around the lesion sites on *dj-lm* leaves, whereas almost no brown spots were detected on wild-type DJ leaves (Fig 1C). At the heading stage, *dj-lm* plants also exhibited a typical senescence phenotype, with withering leaves (Fig 1D). In addition to the cell death and senescence phenotypes, major agronomic traits including plant height, seed setting rate, tiller number, flag leaf angle, 1000-grain weight, and panicle length were affected in the *dj-lm* plants (S1 Table).

### Enhanced resistance of the *dj-lm* mutant to rice blast and bacterial blight pathogens

To determine whether the mutation in *dj-lm* led to enhanced resistance to pathogens, we first inoculated six-week-old DJ and *dj-lm* plants when the *dj-lm* plants displayed lesions with the compatible *Magnaporthe oryzae* isolate RO1-1 using the punch inoculation method. The lesions on *dj-lm* leaves were approximately one-quarter the size of those on DJ leaves (Fig 1E and 1F). Moreover, the relative fungal biomass on *dj-lm* was approximately 13% of that on DJ (Fig 1G). Additionally, we tested the disease response of six-week-old rice plants to the bacterial blight pathogen *Xanthomonas oryzae* pv. *oryzae* (*Xoo*) and found that the disease lesions on *dj-lm* were approximately one-sixth of the length of those on DJ after infection with the *Xoo* isolate PXO99 (Fig 1H and 1I). These results clearly demonstrate that *dj-lm* plants have significantly increased broad-spectrum resistance against both *M*. *oryzae* and *Xoo*.

**Fig 2:**
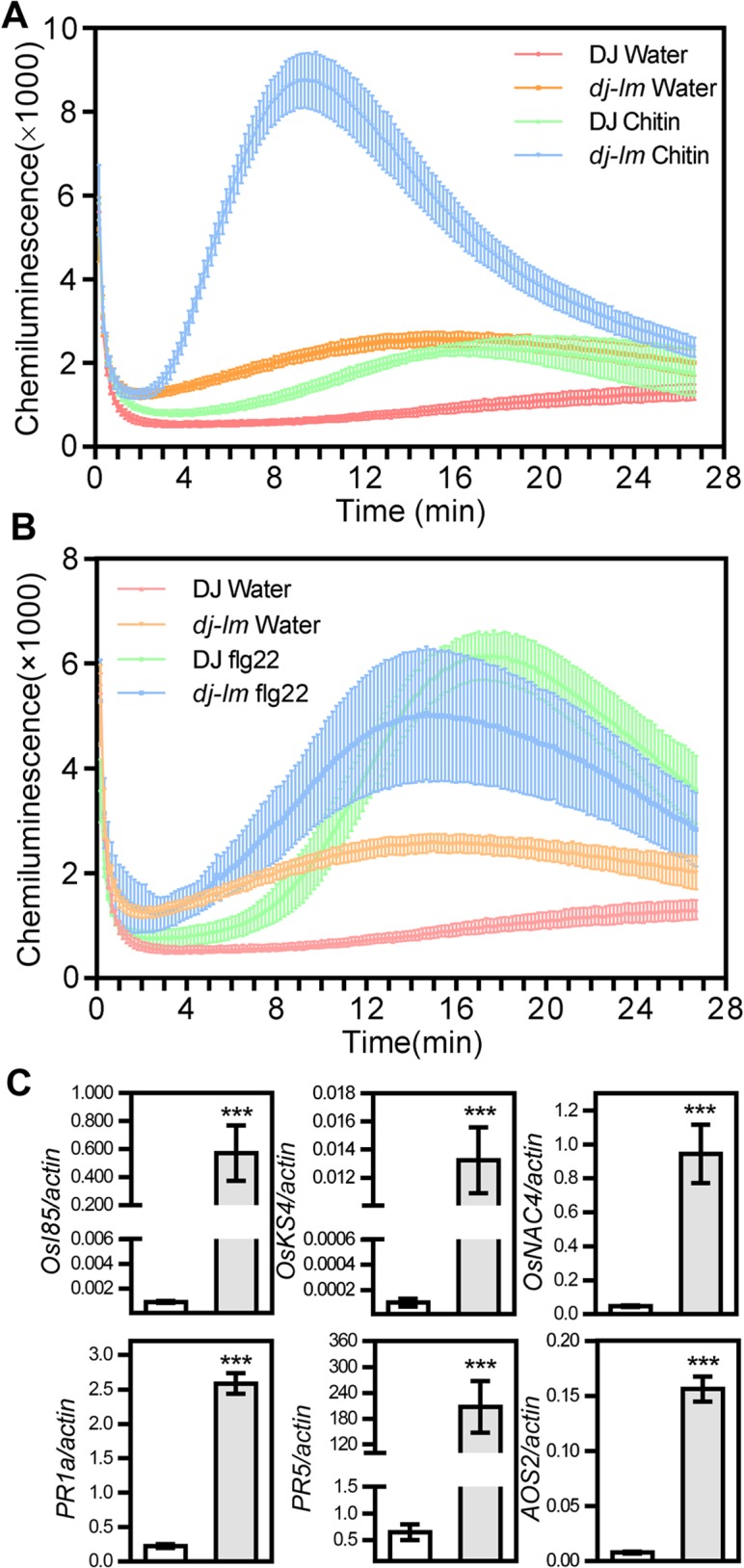
ROS generation and defense-related gene expression in the *dj-lm* mutant. **(A)** ROS bursts of DJ and *dj-lm* after chitin treatment. Values are means ± standard errors of three biological replications. Similar results were obtained from three independent experiments. **(B)** ROS bursts of DJ and *dj-lm* after flg22 treatment. Values are means ± standard errors of three biological replications. Similar results were obtained from three independent experiments. **(C)** Transcript levels of cell death-related and PR genes in DJ and *dj-lm* plants. Values are means and standard errors of three biological replications. White and gray bars represent the transcript levels of the genes tested in DJ and *dj-lm*, respectively. Significance was determined at ***P<0.0001 with a Student’s *t*-test.

ROS generation occurs as an early event in plant cell death [43]. In this study, we used luminol-based chemiluminescence to detect ROS generation in leaf disks from six-week-old plants as reported previously [44]. In the water control as the mock treatment, ROS levels in *dj-lm* were approximately twice those of DJ (Fig 2A), which was consistent with the DAB staining results (Fig 1C). Following chitin treatment, the luminol count in *dj-lm* reached its highest values, roughly 4- to 4.5-fold more than that in DJ, at approximately 10 minutes after chitin application (Fig 2A). However, we could not detect a difference in ROS burst between DJ and *dj-lm* in plants challenged with flg22 (Fig 2B).

To determine whether the transcription of defense-related genes, such as senescence-associated genes, cell death-related genes and pathogenesis-related (PR) genes, was affected in the mutant, we analyzed the expression of these genes from six-week-old DJ and *dj-lm* plants using quantitative RT-PCR. Consistent with the enhanced disease resistance of *dj-lm*, the transcriptional levels of the senescence-associated gene *OsI85*, the cell death-related genes *OskS4* and *OsNAC4* and the PR genes *PR1a*, *PR5*, and *AOS2* were significantly higher in the *dj-lm* plants than in DJ (Fig 2C).

### Map-based cloning of the mutant gene in *dj-lm*

**Fig 3:**
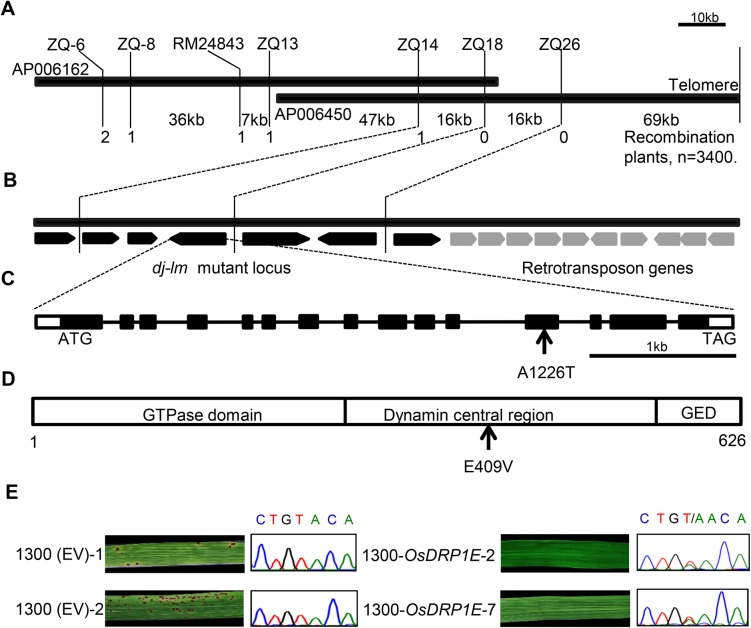
Map-based cloning of *OsDRP1E*. **(A)** Fine physical map of the *dj-lm* candidate locus. The two thick black bars represent PAC clones AP006162 and AP006450. Words above and below the bars indicate SSR markers, InDel markers and the physical distance between the two markers, respectively. The numbers below the maps represent the number of recombination events. (**B)** Predicted ORFs in the *dj-lm* mutant. The thick black bars represent PAC clone AP006450. Arrows indicate the order and orientation of 16 ORFs within the PAC clone AP006450. ORFs in gray are retrotransposon genes. (**C)** Gene structure of *OsDRP1E*. The schematic map shows the coding region (black boxes), the 5’ and 3’ untranslated regions (white boxes) and the intron region (lines). Arrow indicates the mutated nucleotide. **(D)** OsDRP1E protein structure. The three boxes indicate the domains of OsDRP1E. The numbers below the box indicate the size of the protein. Arrow represents the mutated amino acid residue. GED: Dynamin GTPase effector domain. (**E)** Genetic complementation of *OsDRP1E*. Left panel: Leaves from transgenic lines (1300 (EV)-1/2) transformed with the pCAMBIA1300 empty vector and their sequencing chromatograms at the *OsDRP1E* locus. Right panel: Leaves from complemented lines (1300-*OsDRP1E-*2/7) transformed with the pCAMBIA1300-*OsDPR1E* construct and their sequencing chromatograms at the *OsDRP1E* locus.

To isolate the mutant gene that controls the cell death phenotype, we employed a map-based cloning strategy. For the genetic analysis, we crossed *dj-lm* with wild-type DJ and the *indica* cultivar 9311. The F_1_ progenies from the DJ × *dj-lm* and 9311 × *dj-lm* crosses did not have any lesions on their leaves, but the F_2_ populations displayed segregation of the wild-type and lesion mimic phenotypes. The segregation ratio was approximately 3:1 (x^2^<x^2^_0.05_ = 3.84, P>0.05) in both populations, suggesting that the phenotype of *dj-lm* is controlled by a single recessive gene (S2 Table). A total of 3,400 F_2_ recessive individuals from the 9311 × *dj-lm* cross were used for DNA marker and phenotype segregation analysis. The phenotypes and genotypes of recombinant individuals were further confirmed in the F_3_ generation. For the initial mapping, 184 of the 920 pairs of SSR markers from Gramene (http://www.gramene.org) were well distributed on the 12 rice chromosomes and showed polymorphisms between *dj-lm* and 9311. Linkage analysis with the molecular marker and lesion phenotype data in the 9311 × *dj-lm* F_2_ mapping populations delimited the *DJ-LM* candidate gene to a 101-kb genomic region between the InDel marker ZQ14 and the telomere on the long arm of chromosome 9 (Fig 3A). There are 16 putative open reading frames (ORFs) annotated in this genomic region according to the RGAP website (http://rice.plantbiology.msu.edu/) (Fig 3B). Since ten of these ORFs were annotated as retrotransposons, we focused on the six remaining genes (S3 Table). Because we did not detect any difference in the transcript levels of these genes between DJ and *dj-lm* (S2 Fig), we further sequenced a 35-kb genomic region spanning these genes and discovered only one A-to-T nucleotide substitution (Fig 3C). This single-nucleotide polymorphism corresponds to the 1226^th^ nucleotide of the ORF within the LOC_Os09g39960 locus (on the 12th exon), resulting in an amino acid change from E to V at the 409^th^ residue of the annotated protein, OsDRP1E, with a molecular weight of 70 kDa (Fig 3D).

To confirm whether this mutation is responsible for the lesion mimic phenotype, we cloned an 11-kb genomic fragment of LOC_Os09g39960, including a 2,937 bp upstream promoter region, a 4,806 bp coding region, and a 3,047 bp downstream region from DJ into the binary vector pCAMBIA1300. The resulting construct, designated 1300-*OsDPR1E*, was introduced into *dj-lm* calli through Agrobacterium-mediated transformation. We generated 36 individually transformed T_0_ lines and grew them in the greenhouse. None of these plants exhibited lesions, unlike the lesion mimic control plants transformed with the empty vector (EV) (Fig 3E, left panel). To verify that the recovery of the wild-type phenotype was indeed due to the reintroduction of 1300-*OsDPR1E* into the mutant background, we sequenced the mutated *OsDRP1E* region in the two complemented lines, 1300-*OsDRP1E-*2 and -7 (Fig 3E, right panel). The sequencing analysis confirmed that the introgression of the wild type *OsDRP1E* into the mutant background exists in the two complemented lines (Fig 3E, right panel). In addition, we evaluated the disease resistance phenotype and ROS burst in the wild-type DJ, an empty-vector transformed line and two complemented lines. The complemented lines showed the same disease phenotype against *M*. *oryzae* and *Xoo* and ROS burst after chitin treatment as the wild-type DJ (S3 Fig). These results clearly demonstrate that the E409V point mutation causes the lesion mimic phenotype in *dj-lm*.

### Molecular characterization of *OsDRP1E*

Bioinformatics analysis showed that the *DJ-LM* gene encodes a dynamin-related protein, OsDRP1E, comprising three conserved domains: the N-terminal GTPase domain, the dynamin central region, and the dynamin GTPase effector domain (Fig 3D), as determined based on the annotations at NCBI (http://www.ncbi.nlm.nih.gov/cdd). Transcription analysis using RT-PCR revealed that *OsDRP1E* was universally expressed in all rice tissues tested, with relatively high expression in roots and leaves (S4A Fig). As the rice plants grew older, the transcription of *OsDRP1E* generally decreased, but the differences among the investigated growth stages (week 4 to week 14) were not significant (S4B Fig). In addition, the expression level of *OsDRP1E* was not affected by inoculation with the compatible *M*. *oryzae* isolate RO1-1 or the incompatible rice isolate RB22 (S5 Fig).

Phylogenetic analysis of the DRPs from different eukaryotic organisms, including human, yeast, *Arabidopsis*, and rice, revealed that OsDRP1E belongs to the plant DRP1 subgroup (S6A Fig). Moreover, amino acid sequence alignment of the DRPs from various origins revealed high sequence similarity in the DRP central domain, and it demonstrates that E409 in OsDRP1E is one of the most highly conserved amino acid residues in the proteins analyzed (S6B Fig). These results suggest that the residue E409 in OsDRP1E and other DRPs is structurally and functionally important.

### The E409V mutation in OsDRP1E disrupts its self-interaction and the formation of higher-order complexes

**Fig 4:**
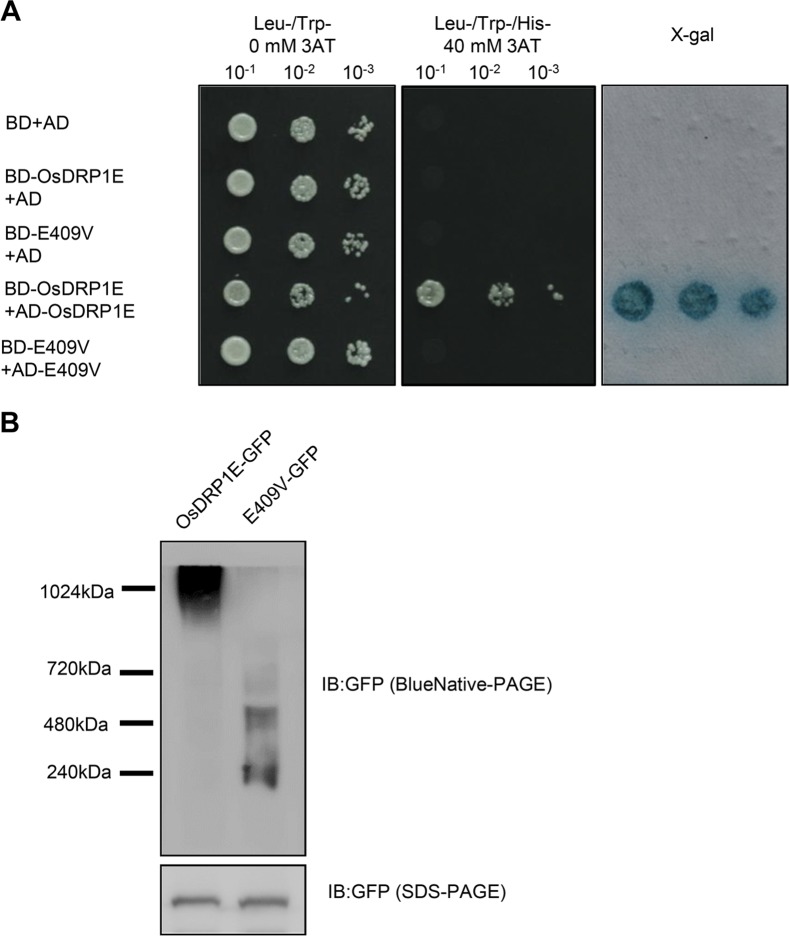
The E409V mutation affects the self-association of OsDRP1E. **(A)** Yeast two-hybrid assays using the *HIS3* reporter to detect the self-interaction of OsDRP1E. Yeast cells transformed with bait and prey constructs as indicated were sequentially diluted 10-fold and plated on synthetic dextrose (SD) medium without Trp, Leu and His amino acids (SD-LTH) and with 0 mM or 40 mM 3-amino-1,2,4,-triazole (3AT), respectively. Yeast cells that either grew in the presence of 40 mM 3AT or were stained blue by X-gal indicate an interaction. **(B)** Immunoblot detection of GFP-tagged OsDRP1E and E409V expressed in *N*. *benthamiana* using Blue Native-PAGE (upper panel) and SDS-PAGE (bottom panel). Blue Native-PAGE followed by immunoblot analysis was used to detect the oligomerization of OsDRP1E-GFP and E409V-GFP. SDS-PAGE followed by immunoblot analysis was used to detect the expression levels of OsDRP1E-GFP and E409V-GFP.

DRPs can form higher-order complexes through self-interaction assembly and the formation of higher-order complexes is a prerequisite for their roles in various cellular processes, such as endocytosis and mitochondrial division [45]. Recent structural studies have revealed that the dynamin central domain plays a vital role in the self-assembly of DRPs [46–48]. We reasoned that the E409V mutation might affect the self-interaction of OsDRP1E based on the observations that the E409 site is located at the self-interaction region and is a highly conserved amino acid residue. To test this hypothesis, we first analyzed the self-interaction of OsDRP1E in the yeast two-hybrid system. As shown in Fig 4A, strong self-interaction was detected in wild-type OsDRP1E, but the mutant OsDRP1E (hereafter referred to as E409V) failed to self-interact in yeast. We then performed native PAGE to examine whether the E409V mutation affects the ability of OsDRP1E to form a higher-order complex. The fusion proteins OsDRP1E-GFP and E409V-GFP were transiently expressed in *N*. *benthamiana* through agro-infiltration. The expression levels of OsDRP1E-GFP and E409V-GFP *in planta* remained similar when analyzed by SDS-PAGE followed by immunoblot detection of GFP (Fig 4B, bottom panel). By contrast, the results of blue native PAGE (BN-PAGE) followed by immunoblot analysis to detect GFP showed that high molecular weight complexes formed from wild-type OsDRP1E *in planta*, while only dimers or tetramers formed from the E409V mutant protein (Fig 4B, upper panel). These results indicate that the E409 residue of OsDRP1E is required for its self-interaction to form higher-order protein complexes.

### The E409V mutation decreases the GTPase activity of OsDRP1E

**Fig 5:**
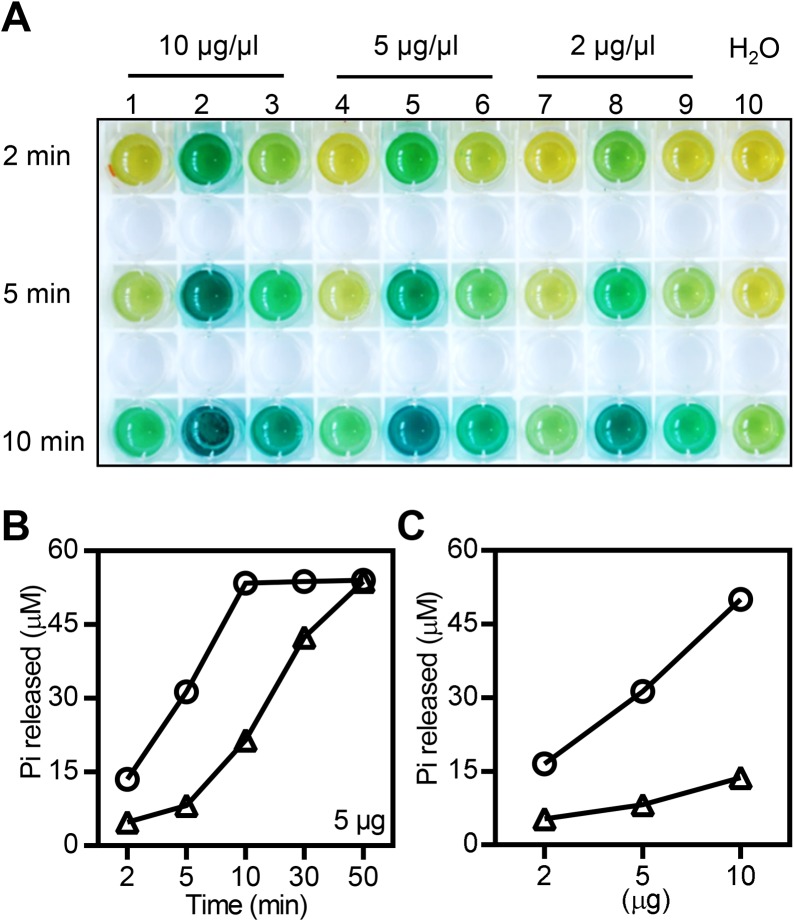
GTPase activity assay of OsDRP1E and E409V. **(A)** GTPase colorimetric reaction of different amounts of MBP-tagged OsDRP1E and E409V or MBP protein at 2, 5, and 10 min. Lanes 1, 4, and 7: MBP protein. Lanes 2, 5, and 8: MBP-OsDRP1E. Lanes 3, 6, and 9: MBP-E409V. Lane 10: H_2_O. **(B**) Pi released in GTP hydrolysis by 5 μg of MBP-tagged OsDRP1E and E409V at different time points, as indicated. Circles represent MBP-OsDRP1E and triangles represent MBP-E409V. Similar results were obtained from two independent experiments. **(C)** Pi released during GTP hydrolysis by 2, 5, and 10 μg of MBP-tagged OsDRP1E and E409V at 5 min. Circles represent MBP-OsDRP1E, triangles represent MBP-E409V. Similar results were obtained from two independent experiments.

DRPs belong to a group of large GTPases with molecular weights above 70 kDa. In contrast to small GTPases, dimerization or higher-order assembly of DPRs promotes the activity of large GTPases and is required for their biological function [47,49]. To determine whether the E409V mutation affects the GTPase activity of OsDRP1E, we examined the *in vitro* GTPase activity of the purified maltose-binding protein (MBP) fusions MBP-OsDRP1E and MBP-E409V (S7A Fig) using a GTPase colorimetric assay. As shown in Fig 5A, no obvious color change was observed in control reactions with the purified MBP protein and H_2_O. Although both MBP-OsDRP1E and MBP-E409V displayed catalytic activity towards the substrate GTP, the former had much stronger activity (Fig 5A). The difference in the activity of these two proteins was confirmed in dosage and time-course assays. Clearly, the levels of phosphates released by MBP-OsDRP1E were significantly higher in both a time-dependent (Fig 5B, S7B Fig) and dosage-dependent manner compared to the E409V mutant (Fig 5C).

### The E409V mutation disrupts the mitochondrial localization of OsDRP1E

Online subcellular localization prediction analysis using the program Euk-mPLoc2.0 specific for plant protein [50] (http://www.csbio.sjtu.edu.cn/bioinf/plant-multi/) and RSLpred specific for rice protein [51] (http://www.imtech.res.in/raghava/rslpred/) showed that OsDRP1E is a mitochondrial protein. To determine the subcellular localization of OsDRP1E, we agro-infiltrated OsDRP1E-YFP and E409V-YFP constructs (S8A Fig) into *N*. *benthamiana* leaves. Immunoblot analysis using anti-YFP antibody showed similar protein levels of OsDRP1E-YFP and E409V-YFP in agro-infiltrated *N*. *benthamiana* leaves (S8B Fig). Confocal microscopy showed spotty, bright fluorescent signals when OsDRP1E-YFP was expressed in *N*. *benthamiana*, while the signal from E409V-YFP was distributed evenly in the cytoplasm, resembling the signal of the YFP control (S9A Fig). To exclude the possibility that the C-terminal fusion of YFP might affect the subcellular localization of OsDRP1E, we also investigated the fluorescence patterns of N-terminal YFP-fused OsDRP1E and E409V *in planta*. As expected, the E409V point mutation abolished the bright speckled signals (S9B Fig).

**Fig 6:**
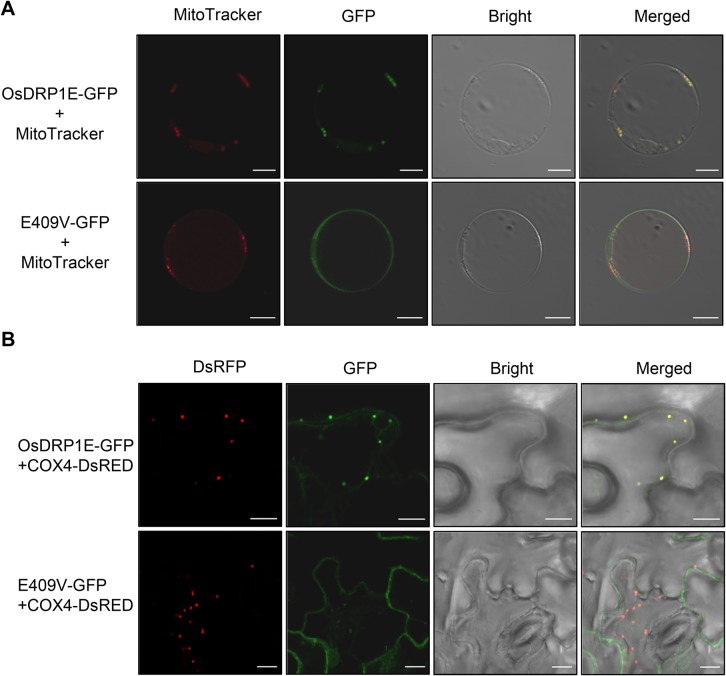
Subcellular localization of OsDRP1E-GFP and E409V-GFP *in planta*. (A) Confocal images of OsDRP1E-GFP and E409V-GFP transiently expressed in rice protoplasts. MitoTracker was used as the mitochondrial marker. Bar = 10 μm. (B) Confocal images of OsDRP1E-GFP and E409V-GFP transiently expressed in *N*. *benthamiana*. Ds-RED-tagged COX4 was used as the mitochondrial marker. Bar = 10 μm.

To verify the subcellular localization of OsDRP1E, we transfected rice protoplasts with the GFP-tagged construct and stained the transfected protoplasts with MitoTracker CMXRos (a mitochondria-specific dye). As shown in Fig 6A, the green fluorescent signals from OsDRP1E-GFP exactly over-lapped the red signals from MitoTracker CMXRos in rice protoplasts. Also, uniform GFP signals were detected in whole cells transfected with E409V-GFP (Fig 6A). We then co-expressed GFP-tagged OsDRP1E and DsRED-tagged COX4, which is a mitochondrial marker protein, in *N*. *benthamiana*, and confirmed that the loss of mitochondrial localization was due to the E409V mutation. Similar differences in GFP signals were observed in the *N*. *benthamiana* cell that co-expressing OsDRP1E-GFP and E409V-GFP (Fig 6B). Taken together, these results demonstrate that OsDRP1E localizes to the mitochondria and that the E409 residue is essential for the mitochondria-specific localization of OsDRP1E.

### The E409V point mutation affects mitochondrial morphology and increases the concentration of cytoplasmic cytochrome *c*

**Fig 7:**
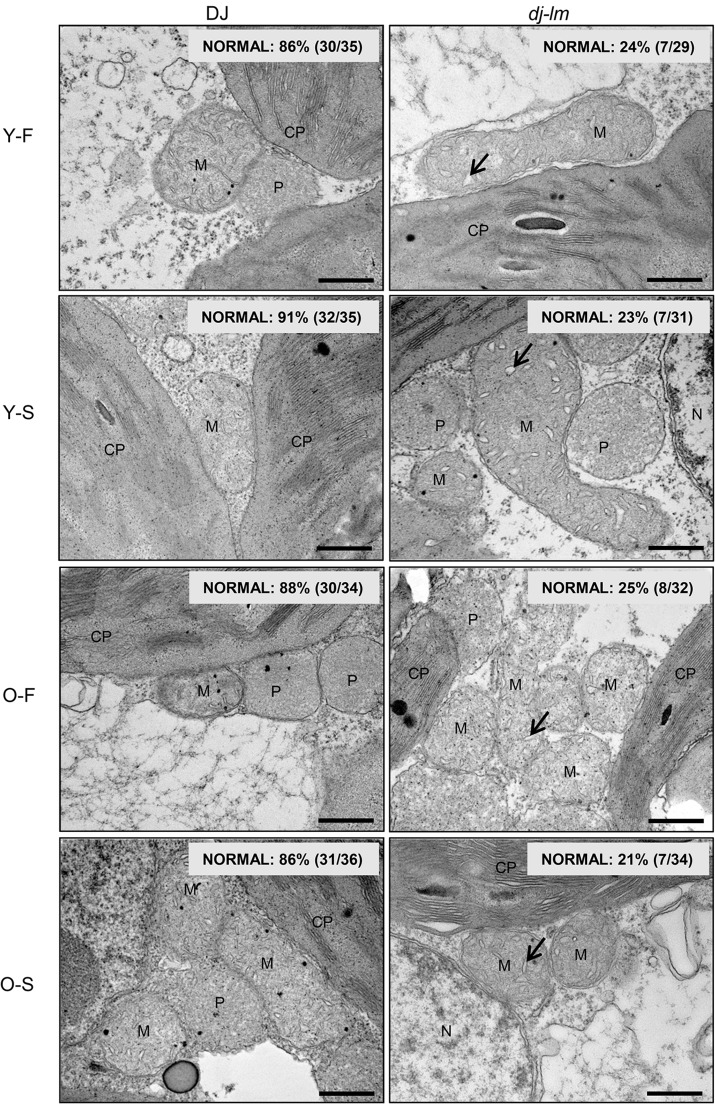
Transmission electron microscopy (TEM) analysis of mitochondrial structure in DJ and *dj-lm* plants. Y: Young leaves from four-week-old plants. O: Old leaves from eight-week-old plants. F and S represent the first and the second leaf from the top. Arrow indicates bubble-like cristae. CP, chloroplast; M, mitochondria; N, nucleus; P, peroxisome. Bar = 0.5 μm.

The discovery that the E409V point mutation abolished OsDRP1E retention in the mitochondria, together with the finding that the mutated protein showed lower GTPase activity, prompted us to investigate whether mitochondrial morphology was affected by the functional loss of OsDRP1E. We observed the ultrastructure of the mitochondria in mesophyll cells (S10 Fig) from four or eight-week-old DJ and *dj-lm* plant leaves by transmission electron microscopy. The *dj-lm* and DJ plants had similar overall number and shapes of mitochondria. However, swelling cristae with vesicle-like structures and reduced intermembrane content were observed in the *dj-lm* mesophyll cells collected from the first and second leaves in four and eight-week-old plants. The ratio of vesicle-like to normal cristae was approximately from 9–14% in DJ compared to 75–79% in *dj-lm* in different growth periods (Fig 7), and the vesicle-like structure of cristae is similar to those in previous reports [52,53].

**Fig 8:**
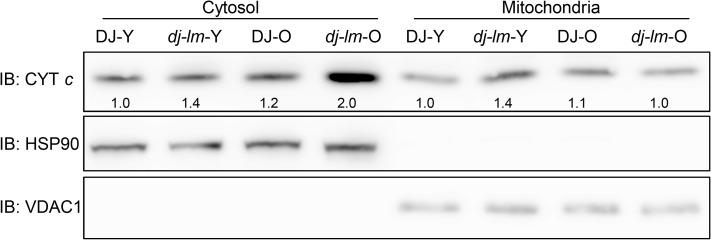
Immunoblot detection of cytochrome *c* in cytosol and mitochondria from DJ and *dj-lm* plants. HSP90 and VDAC1 served as the loading control for cytosolic and mitochondrial protein, respectively. Y: Young leaves from four-week-old plants. O: Old leaves from eight-week-old plants. Numbers below the band in first panel represent the relative cytochrome *c* levels in cytosol and mitochondria as compared to HSP90 and VDAC1, respectively, using the Image J software.

Studies in animals have shown that the release of cytochrome *c* into the cytoplasm induces caspase activity, ultimately leading to PCD [54]. To explore the association of the cytochrome *c* levels and the cell death in rice plants, we isolated subcellular fractions from the plants and determined the cytochrome *c* levels in the cytoplasm and mitochondria using immunoblotting analysis with anti-cytochrome *c* antibody (Fig 8). We found that the increase of the cytochrome *c* levels in the cytoplasm as plants aged from four-week old to eight-week-old. Interestingly, the cytosol cytochrome *c* levels were higher in *dj-lm* than in DJ no matter the mutant plants exhibited cell death lesions or not and reached its maxim values in the *dj-lm* plants after lesion appeared (Fig 8). On the contrary, the mitochondrial cytochrome *c* levels in *dj-lm* remained similar as those in DJ when plants were eight-week-old. To further confirm that the OsDRP1E is indeed required for the cytochrome *c* releasing, we compared the cytochrome *c* level from four-week-old and eight-week-old plants of wild type DJ, empty vector transgenic control (EV) and complemented lines (C2 and C7) (S11 Fig). Similar levels of cytochrome *c* were observed in the DJ and the complemented lines whereas the empty vector control transgenic line (EV) displayed higher protein level of the cytochrome *c* in 4-week or 8-week old plants. Taken together, these results demonstrate that the E409V point mutation in OsDRP1E affects the morphology of the mitochondrial cristae and leads to increased release of cytochrome *c* into the cytoplasm, which may represent the main trigger for the development of the lesion mimic phenotype in *dj-lm* plants.

## Discussion

### The role of OsDRP1E in mitochondria-mediated PCD

In this study, we found that the *dj-lm* mutant displayed a spontaneous cell death phenotype and enhanced resistance to rice blast and bacterial blight pathogens. Using map-based cloning and a genetic complementation approach, we demonstrated that the dynamin-related protein OsDRP1E is a negative regulator of cell death and that the E409V point mutation in OsDRP1E leads to a lesion mimic phenotype. In the past two decades, dynamin and DRPs have been extensively studied in animal and yeast systems. Mutations in DRPs such as OPA1 [55], Mitofusin2 [56], Atlastin [57], and Drp1 [58] have been identified as the causes of many genetic disorders in humans. For example, a lethal mutation (A395D) located at the central domain of Drp1 causes neonatal death in humans [58]. As Drp1 participates in mitochondrial and peroxisomal fission, a nonfunctional mutation of Drp1 leads to the formation of elongated mitochondria [58]. Likewise, mutations at the other conserved residues, G350 and G362, which are also located in the central domain of Drp1, impair the retention of Drp1 on the mitochondria and lead to the production of elongated mitochondria [45]. From a structural point of view, these conserved amino acids, including E409 of OsDRP1E as well as G350, G362, and A395 of Drp1, map to Interface 3, as recently revealed by resolving the crystal structure of the Dynamin-3 tetramer [59]. Interface 3, together with Interface 1, are required for the assembly of tetramers from Dynamin-3 dimers. A series of single-site mutations within Interface 3 of Dynamin-3 yields dimeric proteins, causing deficient liposome binding and reduced GTPase activity. Similarly, our assays of the self-association of E409V-GFP *in planta* showed that the majority of the mutant proteins are dimerized, with fewer tetramers compared to the higher-order complexes formed by wild-type OsDRP1E-GFP. These results suggest that the E409V mutation in OsDRP1E might weaken the polar interactions of the negatively charged amino acid, thereby hampering the formation of higher-order complexes *in planta*. Because the formation of the higher-order complexes promotes the hydrolysis activity of the GTPase domain of dynamin, the E409V mutation affects the GTPase activity of OsDRP1E, as demonstrated in the present study.

In mammals, abnormal mitochondrial cristae are associated with the apoptosis process [60], which in turn causes the release of cytochrome *c* from the mitochondria into the cytoplasm, followed by the induction of caspase-like activity in the cell, ultimately leading to cell death [61]. Mitochondrial outer membrane permeabilization (MOMP) is a crucial event during apoptosis that leads to the release of cytochrome *c*. Studies in HeLa cells have demonstrated that Drp1 plays important roles in regulating MOMP and the morphology of mitochondria [61]. In plants, abnormal mitochondrial cristae trigger changes in MOMP and the release of cytochrome *c* from the mitochondria in early embryonic cells [62]. A previous study revealed the presence of cytochrome *c* in the cytosolic compartment obtained by subcellular protein fractionation followed by western blot analysis [63]. These events also occur during PCD in plants under abiotic stress [64], and they may disrupt ATP biosynthesis, which is dependent on the normal structure of mitochondria [65]. In the current study, we detected abnormal distribution of cytochrome *c* between the mitochondria and cytoplasm in *dj-lm* plants, thus establishing a link between the functional inactivation of OsDRP1E caused by the E409V mutation and mitochondria-mediated cell death, presumably via the release of cytochrome *c* into the cytoplasm. We hypothesis that vesicle-like cristae with increased spaces between the membranes may allow cristae-localized cytochrome *c* to flow freely into the inter membrane space and subsequently to cytosol to trigger cell death activation pathway. However, the exact mechanism of cell death caused by the dysfunction of OsDRP1E requires further investigation. Nevertheless, plants harbor a known PCD pathway controlled by Type I meta-caspases such as AtMC1 and AtMC2 [66]. Therefore, we speculate that a PCD pathway exists in plants that is mediated by the mitochondria through a cytochrome *c*-caspase-like activation pathway (as in animals) and that cytochrome *c* might act epistatically on the meta-caspases. To determine whether cytochrome *c* induces cell death in plants, we infiltrated different concentrations of cytochrome *c* into *N*. *benthamiana* leaves and observed phenotypes at different time points after infiltration. We did not see any obvious cell death phenotype in the treated leaves. We speculate that exogenous cytochrome *c* may be not able to cross the plasma membrane or be inhibited in an inexplicit mechanism in plant cells to activate the caspase-like pathway as in animal cells. Therefore, more research is needed to investigate the function of cytochrome *c* in plant PCD.

### The role of OsDRP1E in mitochondrial membrane structure

In *Arabidopsis*, DRP3A and DRP3B are the closest homologs to human Drp1 and are functionally redundant during mitochondrial fission. The null mutants *drp3a* and *drp3b-1* have mitochondria that are slightly longer than those of wild-type plants, while *drp3a/drp3b-1* double mutant has mitochondria that form an extremely elongated, interconnected network structure [67]. However, in this study, we did not obtain direct evidence that OsDRP1E is responsible for mitochondrial fission, as no elongated mitochondria were found in *dj-lm*. This result might be due to the functional redundancy between OsDRP1E and OsDRP1C or OsDRP1D, as phylogenetic analysis revealed that these three DRPs belong to a close clade. Nevertheless, the observation of an abnormally high percentage of bubble-like structures of mitochondrial cristae in *dj-lm* plants, together with the loss of mitochondrial localization of the mutant protein OsDRP1E-E409V, strongly suggest that OsDRP1E participates in the maintenance of mitochondrial membrane structures.

### Subcellular localization of OsDRP1E

The functionally divergent DRPs can target various organelles in plants [36,66]. These studies helped reveal the functional divergence of this group of multifaceted proteins. Using confocal microscopy, we demonstrated that OsDRP1E is localized to mitochondria based on the following observations. First, OsDRP1E tagged with YFP or GFP at either the N- or C-terminus produced similar speckled patterns in *N*. *benthamiana*, while YFP- and GFP-tagged E409V mutant proteins did not produce these specific, speckled patterns. Second, when we used the mitochondria-specific dye MitoTracker or the marker protein COX4 as an indicator, the florescent signals from OsDRP1E-GFP co-localized with these mitochondrial markers. The observation that E409V leads to targeting of the protein to the cytosol might be due to the inability of OsDRP1E to form polymers at the mitochondrial membrane. Finally, the differences in localization patterns between OsDRP1E and E409V matched the functional consequences of the mutation, as revealed by the changes in mitochondrial morphology observed by transmission electron microscopy. However, it is currently unclear whether the targeting of OsDRP1E to the mitochondria occurs via recruitment by unknown adapter proteins or through direct association with the mitochondrial membrane. Therefore, identifying and characterizing interacting proteins of OsDRP1E will provide new insights into the OsDRP1E-mediated regulation of PCD in rice.

### Function of OsDRP1E in rice immunity

Apart from physical barriers, ROS burst is the first layer of defense in plant PTI signaling [68]. Mitochondria play an important role in mediating the balance of ROS levels in plant cells. Because *OsDRP1E* may participate in the maintenance of mitochondrial membrane structures, mutation of the gene might cause structure changes and elevated ROS levels. Indeed, we detected higher ROS levels in *dj-lm* plants than in wild type, even in the absence of any treatment, suggesting the existence of basal-level activation of the defense pathway in *dj-lm*. Interestingly, the ROS levels were significantly higher in *dj-lm* plants after chitin treatment, while there was no difference between the ROS bursts detected in DJ and *dj-lm* after flg22 treatment. These results suggest that OsDRP1E-induced ROS generation is limited to the chitin-signaling pathway.

Most lesion mimic mutants display enhanced disease resistance [23,69] and significant up regulation of defense-related genes such as: *PR1a* and *PR5*, marker genes associated with defense-related responses in rice [70,71]; *OsKS4* and *AOS2*, encoding important biosynthetic enzymes in the phytoalexin and jasmonic acid biosynthesis pathways, respectively [72,73]; *OsNAC4*, encoding a protein that participates in the induction of HR cell death and may regulate the transcription of multiple genes, including *OsHSP90* and *IREN* [74], and *Osl85*, a senescence-associated gene that functions in fatty acid metabolism [75]. In summary, the higher transcript levels of these defense-related genes and senescence-associated genes correlate well with the enhanced resistance to rice blast and bacterial blight pathogens and the senescence phenotypes observed in *dj-lm* plants.

## Methods

### Plant growth conditions and agronomic trait measurements

Rice cultivars DJ (*Oryza sativa* ssp. *japonica*) and 9311 (*Oryza sativa* ssp. *indica*) were used in this study. Rice plants were cultured in a growth chamber at 26/22°C under a 14 h light/10 h dark cycle or in a paddy field on our experimental farm in June through October. Agronomic traits of rice plants grown in the paddy field were measured, including plant height, seed setting rate, tiller number, flag leaf angle, 1000-grain weight, and panicle length.

### Trypan blue and DAB staining

Leaves from *dj-lm* plants containing lesions and leaves from DJ at the same growth stage (eight-week-old) were submerged in lactic acid-phenol-Trypan blue solution (0.25% Trypan blue, 25% lactic acid, 23% water-saturated phenol, and 25% glycerol) for staining, as previously described [69]. Briefly, the leaf samples were incubated in a boiling water bath for 10 min, cooled to room temperature and incubated in the Trypan blue staining solution supplemented with chloral hydrate (0.25%) for 48 h.

DAB staining was used to detect H_2_O_2_ accumulation in the leaves as described previously [76]. Briefly, the leaves of eight-week-old rice plants were submerged in DAB solution (0.1%, pH3.8) at 26°C for 8 h in the light. After draining off the DAB solution, the leaves were boiled for 10 min in a water bath containing 95% ethanol for destaining, followed by incubation in 95% ethanol at room temperature.

### Rice blast and bacterial blight inoculations

The leaves were subjected to punch inoculation to measure the rice blast resistance of DJ and *dj-lm* plants using *M*. *oryzae* isolate RO1-1, as previously described [44]. Briefly, six to eight week-old leaves were lightly wounded using a mouse ear puncher, and 7 μl of spore suspension (5×10^5^ spore· ml^-1^) was added to the wound site, which was then sealed in a small chamber with transparent tape. The inoculated plants were incubated in the dark for 24 h in a growth room at 28°C with 100% relative humidity, and then moved to a growth chamber at 26/22°C under a 14 h light/10 h dark cycle with 80% relative humidity. Disease symptoms and fungal biomass in the infected leaves were surveyed 7 d after inoculation. The fungal biomass in the infected leaf tissue was quantified using the method was described in a previous study [44]. Briefly, the infected rice tissue about 3 × 1cm was cut for DNA extraction using the CTAB method. After RNase A treatment, DNA- based qPCR was performed using Bio-Rad iQ2 PCR system (Bio-Rad). The threshold cycle value (C_T_) of *M*. *oryzae Pot2* gene against the C_T_ of rice *Os-Ubq* gene was used to calculate the relative fungal biomass in rice leaves. The C_T_ of *Os-Ubq* was subtracted from the CT of *Pot2*, and then, using the equation E^CT (*Os-UBQ*)–CT (*Mo-Pot2*)^ that represents the ratio of (*Mo-Pot2*/*Os-Ubq*) to calculate the relative fungal biomass, in which the amplification efficiency, E, is 2 for the primer pairs designed for the respective genes.

The leaf-clipping method was used to measure the bacterial blight resistance of DJ and *dj-lm* plants using the isolate PXO-99 in greenhouse-grown plants as described previously [77]. Briefly, tips of the top-two fully expanded leaves of eight-week-old DJ and *dj-lm* which showed lesion mimics were cut with scissors and inoculated with *Xoo* isolate PXO99 solution (OD_595_ = 0.5). The inoculated plants were moved to greenhouse at 28°C, 12/12 h light/dark photoperiod. The lesion length was measured at 14 d after inoculation.

### Detection of ROS bursts

Leaf disks were excised from the fully expanded leaves (the second or third leaf from the top) of six to eight-week-old plants using an ear-hole puncher and floated on sterile distilled water overnight. Three leaf disks were placed in a 1.5 ml microcentrifuge tube containing 100 μl of luminol (Bio-Rad Immun-star horseradish peroxidase substrate 170–5040), 1μl of horseradish peroxidase (Jackson Immuno Research) and 100 nM flg22 or 8 nM hexa-*N*-acetyl-chitohexaose, with sterile distilled water for the control. The tube was immediately placed in a Glomax 20/20 luminometer (Promega) and the luminescence was recorded at 15 s intervals for 30 min.

### Transcriptional analysis using RT-PCR

Total RNA was extracted with Trizol reagent (Invitrogen) according to the manufacturer’s protocol. After DNaseI treatment, 2 μg of RNA was added to a 20 μl reaction system to synthesize first-strand cDNA using the Reverse Transcription System (Promega) according to the manufacturer’s instructions. Using 1.0 μl of 1:10 diluted cDNA as template, PCR was performed in a 20 μl reaction volume with Bio-Rod SYBRII Super-Mix buffer on a Bio-Rad iQ2 PCR system (Bio-Rad). The rice *actin* gene was used as the internal control. Gene-specific primers for PCR are listed in S4 and S5 Tables.

### Map-based cloning of *OsDRP1E*

Genetic analysis was performed using 133 individuals from the F_2_ population of the *dj-lm* × DJ cross and 126 individuals from the F_2_ population of the *dj-lm* × 9311 cross. F_2_ recessive individuals from the *dj-lm* × 9311 cross were used for DNA marker and phenotype segregation analyses. The phenotype and genotype of each recombinant individual was confirmed in the F_3_ generation. For the initial mapping, SSR markers from Gramene (http://www.gramene.org) were used for linkage analysis. For fine-mapping of the candidate *dj-lm* mutant gene, InDel markers were developed based on the sequence differences between the *japonica* variety NPB (http://rgp.dna.affrc.go.jp/) and the *indica* variety 9311 (http://rise2.genomics.org.cn/page/rice/index.jsp). The primers used for fine mapping are listed in S4 Table. The PCR products were separated by electrophoresis in 8% polyacrylamide gels or 3% agarose gels depending on the amplicon size. For complementation tests, the wild-type *DJ-LM* genomic DNA fragment was cloned into binary vector pCAMBIA1300. This derivative construct or the empty vector was mobilized into Agrobacterium stain EHA105 by electroporation and used to transform the *dj-lm* mutant. The transformants were grown in a growth chamber for phenotypic and genotypic investigations.

### Phylogenetic analysis

Alignment of the DRP amino acid sequences was performed using CLUSTAL W with DRP amino acid sequences obtained from NCBI (blast.ncbi.nlm.nih.gov/Blast.cgi).The phylogenetic trees were constructed by the neighbor joining method [78] using MEGA 6.06 software.

### Yeast two-hybrid assay

The ProQuest yeast two-hybrid system (Invitrogen) was used to screen the OsDRP1E-interacting proteins according to the product manual. The coding sequence of *OsDRP1E* and *OsDRP1E (E409V)* were cloned into bait vector pDBleu and prey vector pPC86, respectively. The bait and prey vectors were co-transformed into yeast strain MAV203 and the transformants were selected on synthetic dextrose medium without Leu and Trp (SD-Leu-Trp). The single transformed yeast was subjected to 10-fold serial dilutions and plated on SD-Leu-Trp-His medium including 0 or 40 mM 3-amino-1, 2, 4-triazde (3AT, Sigma-Aldrich). Three independent experiments were performed, and positive clones on the SD-Leu-Trp-His plates were stained with 2.5 mM X-gal to detect β-galactosidase activity.

### Agroinfiltration in *N*. *benthamiana*, protein preparation, and immunoblot analysis

The fusion constructs of OsDRP1E and E409V with GFP or YFP were transformed into Agrobacterium strain EHA105 via electroporation. Six-week-old *N*. *benthamiana* leaves were infiltrated with EHA105 transformants containing the appropriate constructs as described previously [44]. After infiltration for 48 h, the leaf samples were collected for confocal microscopy and immunoblot analysis. Confocal microscopy was performed using a Zeiss LSM710 confocal laser-scanning microscope. For immunoblot analysis, 100 mg fresh *N*. *benthamiana* leaf samples were finely ground in liquid nitrogen and combined with 100 μl 2 × loading buffer (10% glycerol, 50 mM Tris-Cl [pH6.8], 2% β-mercaptoethanol, 0.02% bromophenol blue, 2% SDS). After boiling for 5 min in a water bath and centrifugation for 5 min at 13,000 rpm at room temperature, 15 μl of the supernatant was loaded onto an SDS-PAGE gel for immunoblot analysis using anti-GFP and anti-YFP antibody (1:5000 anti-GFP/anti-YFP dilution, Roche).

### Blue native (BN) PAGE

Blue native PAGE was performed as described previously [64]. Briefly, 100 mg samples of fresh of *N*. *benthamiana* leaves that transiently expressed the OsDRP1E-GFP or E409V-GFP fusion proteins were ground in liquid nitrogen using a mortar and pestle. A Native PAGE Sample Prep Kit (Invitrogen) was used to isolate the native tobacco proteins. The ground samples were combined with 400 μl 1 × Tris-buffered saline buffer (2% Triton X-100), vortexed, and incubated on ice for 30 min. The homogenates were centrifuged twice at 17,000 g, 4°C, 20 min per centrifugation. Then, 25 μl of the supernatant was transferred to a new tube, combined with 3 μl of 5% Coomassie Brilliant Blue G250 and separated in a 4 to 16% native PAGE gel (Invitrogen) according to the manual. Immunoblot analysis was performed using anti-GFP antibody (1:5000). Chemiluminescence was detected using an Image Quant LAS 4000.

### Fusion protein purification and GTPase activity assay

Fusion constructs of MBP-OsDRP1E and MBP-E409Vwere transformed into *E*. *coli* BL21 (DE3) for protein expression. BL21 was grown at 28°C to OD600 of 0.6, and IPTG was added to a final concentration of 2 mM, followed by incubation for 6–8 h at 28°C. The cell pellets were harvested by centrifugation for 15 min at 5000 g, 4°C and resuspended in 1/10 volume bacterial culture in 0.5 M Tris-HCl buffer (pH8.0) containing protease inhibitor cocktail (Roche). After sonication, the lysates were centrifuged for 10 min at 15,000 g, 4°C. Protein purification was performed using an Amylose resin (NEB, E8021) column according to the product manual. GTPase activity was determined using a GTPase Assay Kit (Innova Biosciences, 602–0120). The enzyme activity was determined based on the amount of phosphate released during GTP hydrolysis, which was calculated according to a phosphate standard curve prepared using the 0.1 mM phosphate stock included in the GTPase Assay Kit.

### Rice protoplast isolation and transient expression

Rice protoplast isolation and transfection were performed as described previously [79]. Briefly, 1 μg of plasmid was transfected into rice protoplasts using the polyethylene glycol 4000 (PEG4000)-mediated transfection method. MitoTracker staining was performed according to the product manual. Briefly, the protoplasts were incubated in 200 nM MitoTracker CMXRos (Invitrogen) in W5 buffer (154 mM NaCl, 125 mM CaCl_2_, 5 mM KCl, 2 mM MES, pH5.6) for 30 min at room temperature and washed three times in W5 buffer. Fluorescence images were taken under a Zeiss LSM710 confocal laser-scanning microscope at 559 nm excitation and 560 nm emission.

### Transmission electron microscopy

Four-week-old and eight-week-old rice leaves (same region in the first and second leaves from the top and three biological leaf samples were taken) were cut into 1 mm^2^ sections and submerged in 2.5% glutaraldehyde in sodium phosphate buffer (pH7.2) for 4 h at 4°C. The samples were prepared as previously described [24]. The images were observed under a transmission electron microscope (H-7650B, Hitachi LTD). To quantify the abnormal mitochondria in the mutant and WT plants, we counted approximate thirty mitochondria in each sample. A mitochondrion is considered abnormal when the ratio of vesicle-like to normal cristae is more than 50% and a mitochondrion is considered normal when the ratio of vesicle-like to normal cristae is less than 50%.

### Detection of cytochrome *c* contents in rice plants

The mitochondria were isolated using the plant mitochondrial extraction kit (Biohao Biotechnology Co. #P0045) according to the product manual. Briefly, 200 mg samples of fresh rice leaves were ground in liquid nitrogen using a mortar and pestle. The ground samples were combined with 1,000 μl of cold lysis buffer (0.5%β-mercaptoethanol), and vortexed. The homogenates were centrifuged for 10 min at 1,000 g, 4°C. The supernatant, containing cytoplasmic and mitochondrial proteins, was transferred to a new tube and centrifuged for 10 min at 16,000 g at 4°C. The supernatant was collected as the cytoplasmic protein fraction, and the pellet was washed in 500 μl washing buffer and was centrifuged for 5 min at 1,000 g at 4°C. The supernatant from the washing buffer was centrifuged for 10 min at 16,000 g at 4°C. The mitochondrial protein fraction pellet was dissolved using 100 μl store buffer. The cytoplasmic and mitochondrial protein fractions were mixed with the same volume of 2 × loading buffer, respectively, and incubated in a boiling water bath for 5 min. The mixed samples were loaded onto a SDS-PAGE gel for immunoblot analysis using the following antibodies at the appropriate dilutions: 1:8000 anti-cytochrome *c*, 1:6000 anti-VDAC and 1:6000 anti-HSP90 (Agrisera). Chemiluminescence was detected using an Image Quant LAS 4000 and the software Image J was used to measure the relative protein levels.

### Gene cloning and plasmid construction

The OsDRP1E/E409V-GFP, OsDRP1E/E409V-YFP and YFP-OsDRP1E/E409V fusion constructs were generated for the subcellular localization and BN-PAGE experiments. The full-length cDNAs of *OsDRP1E* and *OsDRP1E* (*E409V*) containing an ORF without the stop codon were amplified with primers 1-F/R including the *Sma*I and *Kpn*I restriction sites, and the PCR product was inserted into pYBA-1132 (-GFP), pYBA-1155 (-YFP), and pYBA-1135 (YFP-), respectively, after double digestion with *Sma*I and *Kpn*I. For the yeast two-hybrid interaction assay, the coding sequence of *OsDRP1E* and *OsDRP1E* (*E409V*), including the *Sma*I and *Spe*I restriction sites, were amplified with primers 1-F/primer2-R. The PCR product was inserted into bait vector pDBleu or prey vector pPC86, respectively, after double digestion with *Sma*I and *Spe*I. For the GTPase activity assay, the coding sequence of *OsDRP1E* and *OsDRP1E* (*E409V*), including the *BamH*I and *Sal*I restriction sites, were amplified with primer3-F/R, and the PCR product was inserted into vector pMalC2 after double digestion with *BamH*I and *Sal*I. The fusion plasmid was transformed into *E*. *coli* strain DE3 for the OsDRP1E protein GTPase activity assay. For the complementation test, the 5’ terminal portion and 3’ terminal portion of the *OsDRP1E* genomic fragments were amplified using primers BQ5-2R-kpnI/BQ4-1F and BQ4-3F/BQ3-1F-SalI, respectively. The fragments with the correct sequences were sub cloned (via two steps) into pCAMBIA1300 using a combination of *Kpn*I/*Sal*I and *Kpn*I digestion. The primer information is listed in S6 Table.

### Accession numbers

Sequence data from this work can be found in the Rice Genome Annotation Project or GenBank database under the following accession numbers and GI numbers: OsDRP1A (AK065908), OsDRP1B (AK072230), OsDRP1C (AK061703), OsDRP1D (AK073186), OsDRP1E (AK069270), OsDRP2A (AK102187), OsDRP2C (AK069134), OsDRP3A (AK073965), OsDRP3B (AK105435), OsDRP3C (AK111167) from rice; AtDRP1A (NP_851120), AtDRP1B (NP_191735), AtDRP1C (NP_172936), AtDRP1D (NP_850420), AtDRP1E (NP_567094) from *Arabidopsis*; Drp1 (NP_036193) from *H*. *sapiens*; Dlp2 (AAF51235) from *D*. *melanogaster*; Drp1 (AAL56621) from *Caenorhabditis elegans*; Dlp (Q09748) from *Schizosaccharomyces pombe*; Dnm (AAA99998) from *Saccharomyces cerevisiae*. PYBA-1132 (KF876796); pYBA-1135 (KF876799); pYBA-1155 (KF876807).

## Supporting Information

S1 Fig Representative leaves of DJ and *dj-lm* plants grown in the field. (TIF)

S2 Fig Transcriptional analysis of six *DJ-LM* candidate genes between Dj and *dj-lm*. (TIF)

S3 Fig The resistance phenotypes and ROS burst of the wild type DJ, transgenic plants EV (pCAMBIA1300 empty vector) and, C2/7 (pCAMBIA1300-*OsDRP1E-*2/7). (TIF)

S4 Fig Spatial and temporal transcription analysis of *OsDRP1E*. (TIF)

S5 Fig Time-course transcriptional analysis of *OsDRP1E* between compatible (inoculated with isolate Ro1-1) and incompatible reaction (inoculated with isolate RB22) in DJ. (TIF)

S6 Fig Sequence analysis of DRP proteins from diverse organisms. (TIF)

S7 Fig GTPase activity assay of OsDRP1E and OsDRP1E (E409V). (TIF)

S8 Fig Expression of YFP-tagged fusion protein OsDRP1E and E409V in *N*. *benthamiana* leaves. (TIF)

S9 Fig Subcellular localization of OsDRP1E-GFP and E409V-GFP *in planta*. (TIF)

S10 Fig The cross section of rice leaves under TEM. (TIF)

S11 Fig Immunoblot detection of cytochrome *c* in cytosol and mitochondria from the wild type DJ, transgenic plants EV (pCAMBIA1300 empty vector) and C2/7 (pCAMBIA1300-*OsDRP1E-*2/7). (TIF)

S1 Table Agronomic traits of DJ and *dj-lm*. (DOCX)

S2 Table Genetic analysis of F_2_ populations. (DOCX)

S3 Table Information about candidate genes. (DOCX)

S4 Table Primers used for fine mapping. (DOCX)

S5 Table Primers used for transcriptional analysis. (DOCX)

S6 Table Primers used for functional analysis of *OsDRP1E*. (DOCX)
