# Supplementary figures and images for: The Rice Dynamin-Related Protein OsDRP1E Negatively Regulates Programmed Cell Death by Controlling the Release of Cytochrome c from Mitochondria
Source: PLoS Pathog. 2017 Jan 12;13(1):e1006157. doi: 10.1371/journal.ppat.1006157 (PMC5266325; doi:10.1371/journal.ppat.1006157)

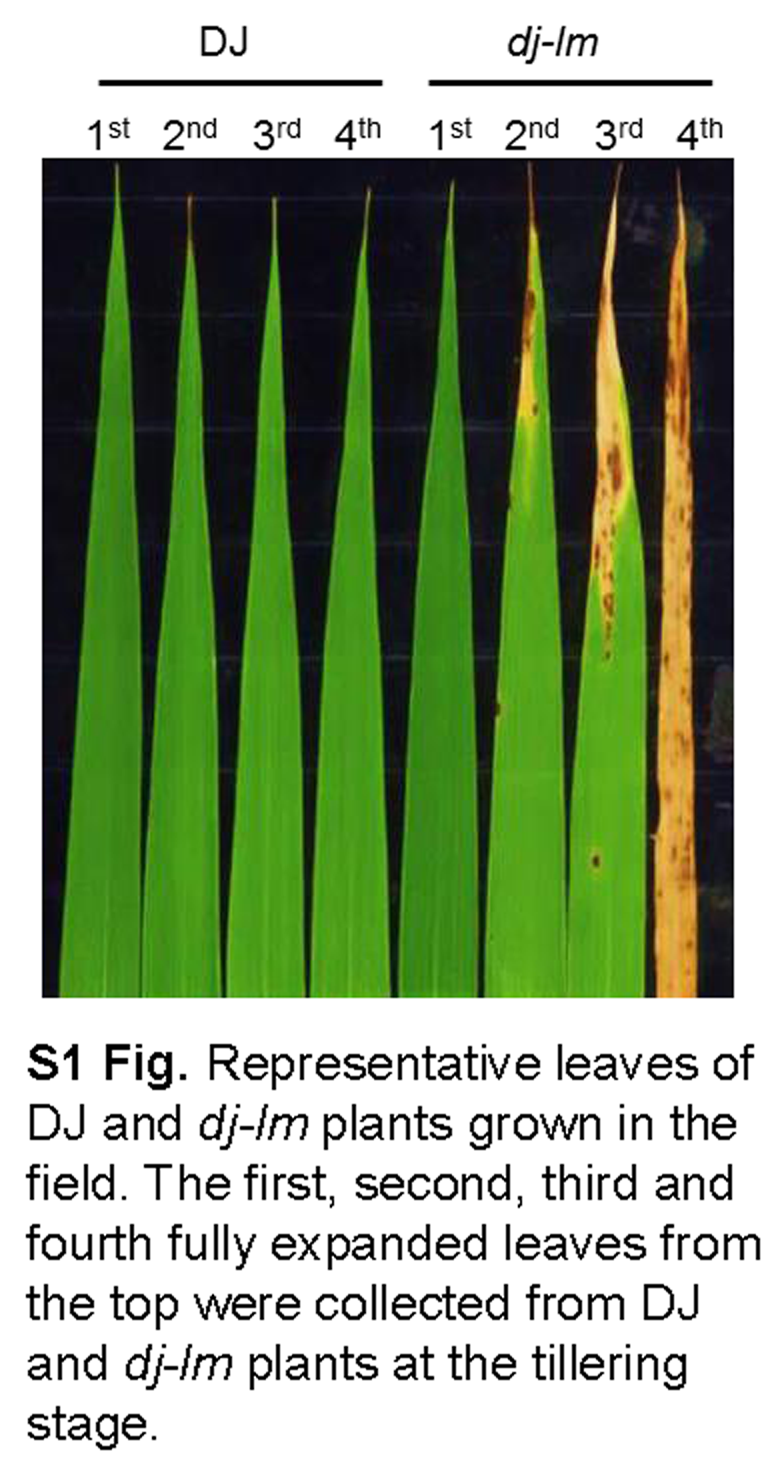

Supplement: S1 Fig — (TIF) [file ppat.1006157.s001.tif]

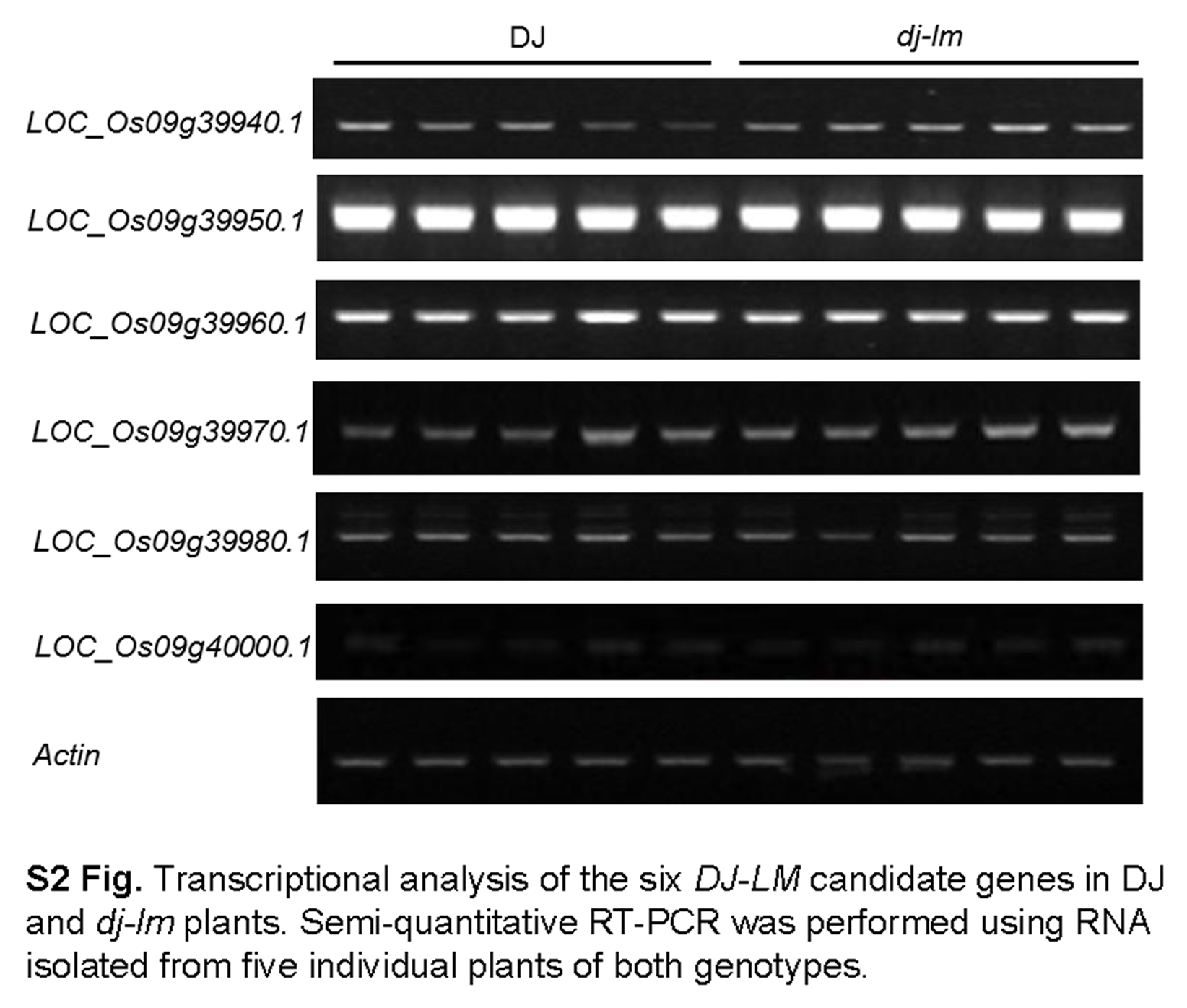

Supplement: S2 Fig — (TIF) [file ppat.1006157.s002.tif]

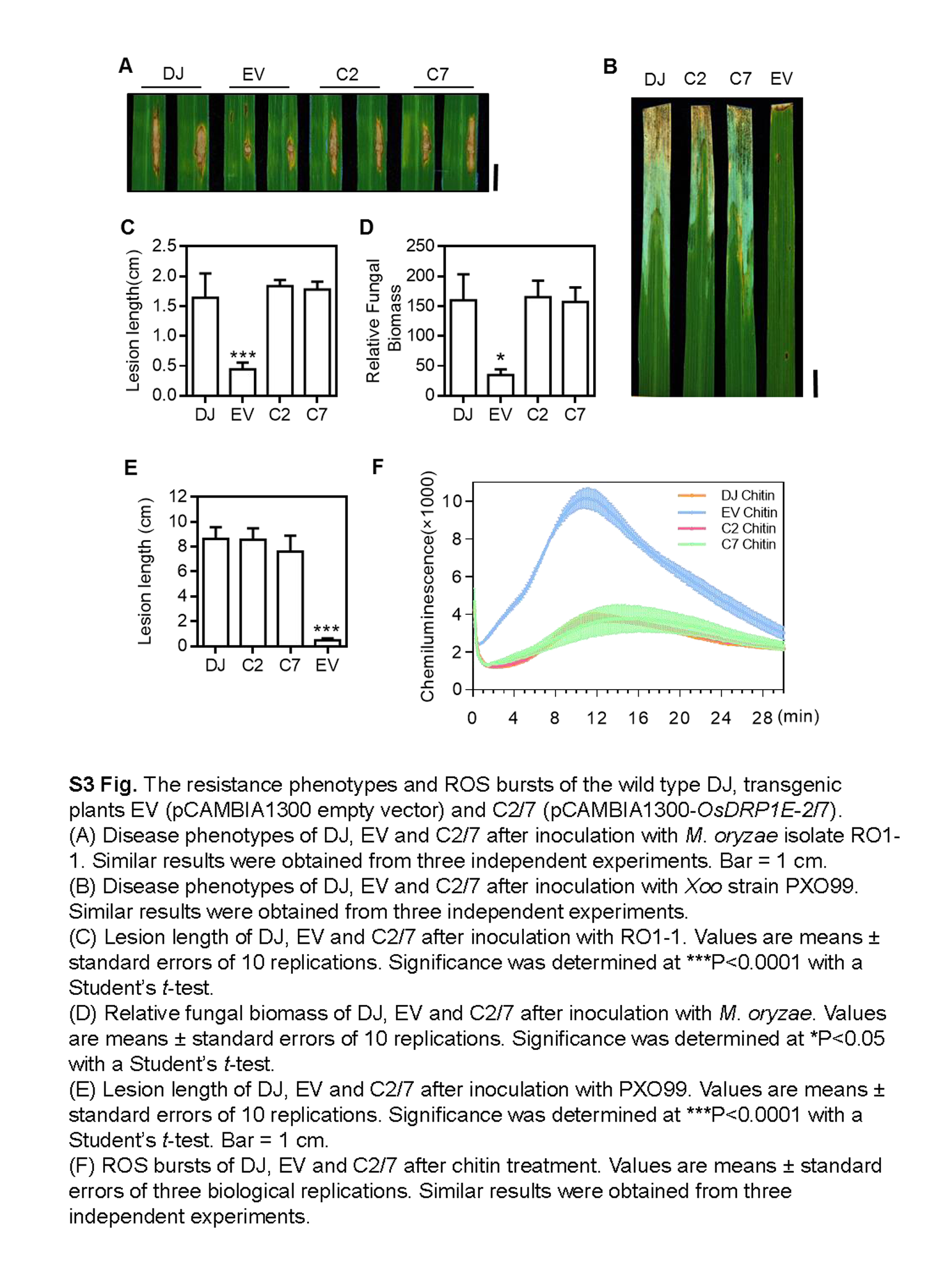

Supplement: S3 Fig — (TIF) [file ppat.1006157.s003.tif]

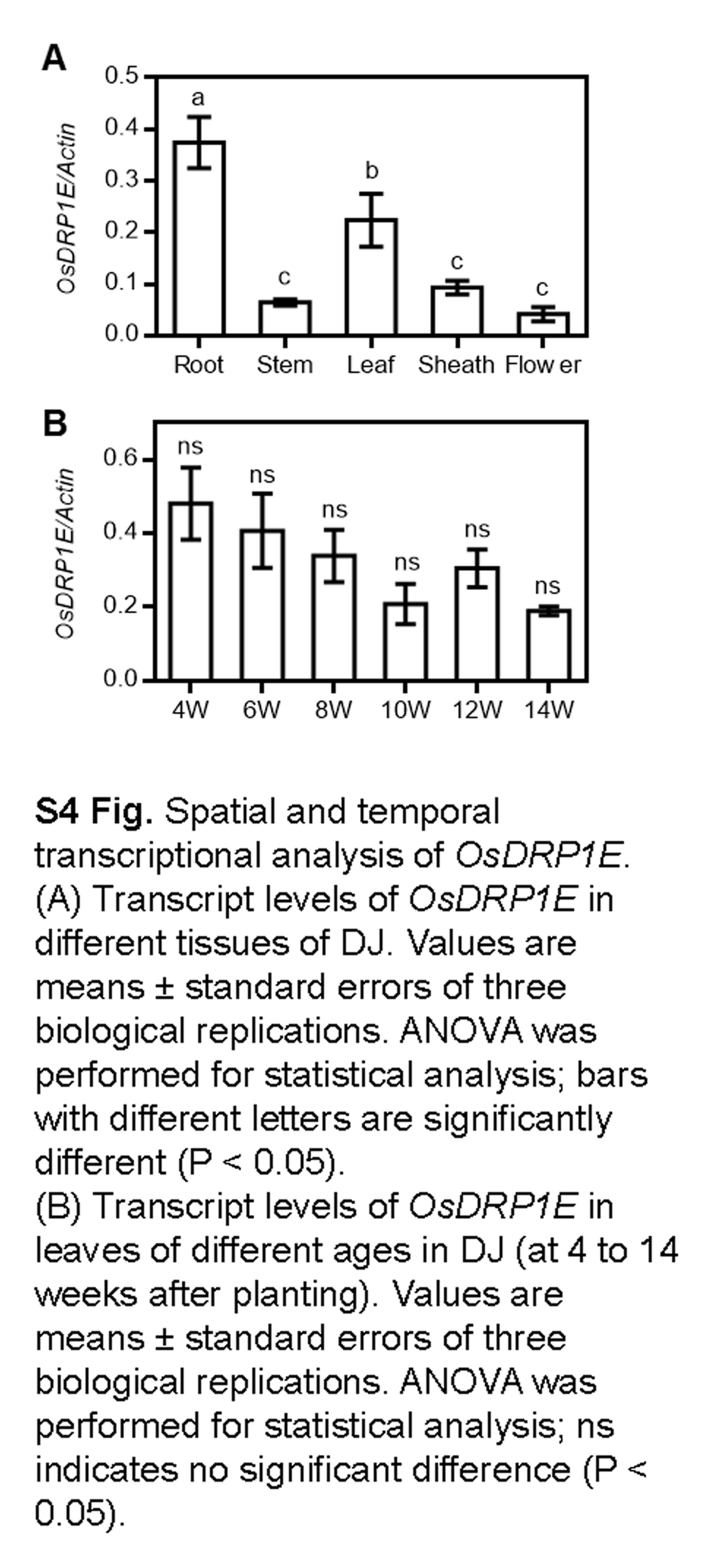

Supplement: S4 Fig — (TIF) [file ppat.1006157.s004.tif]

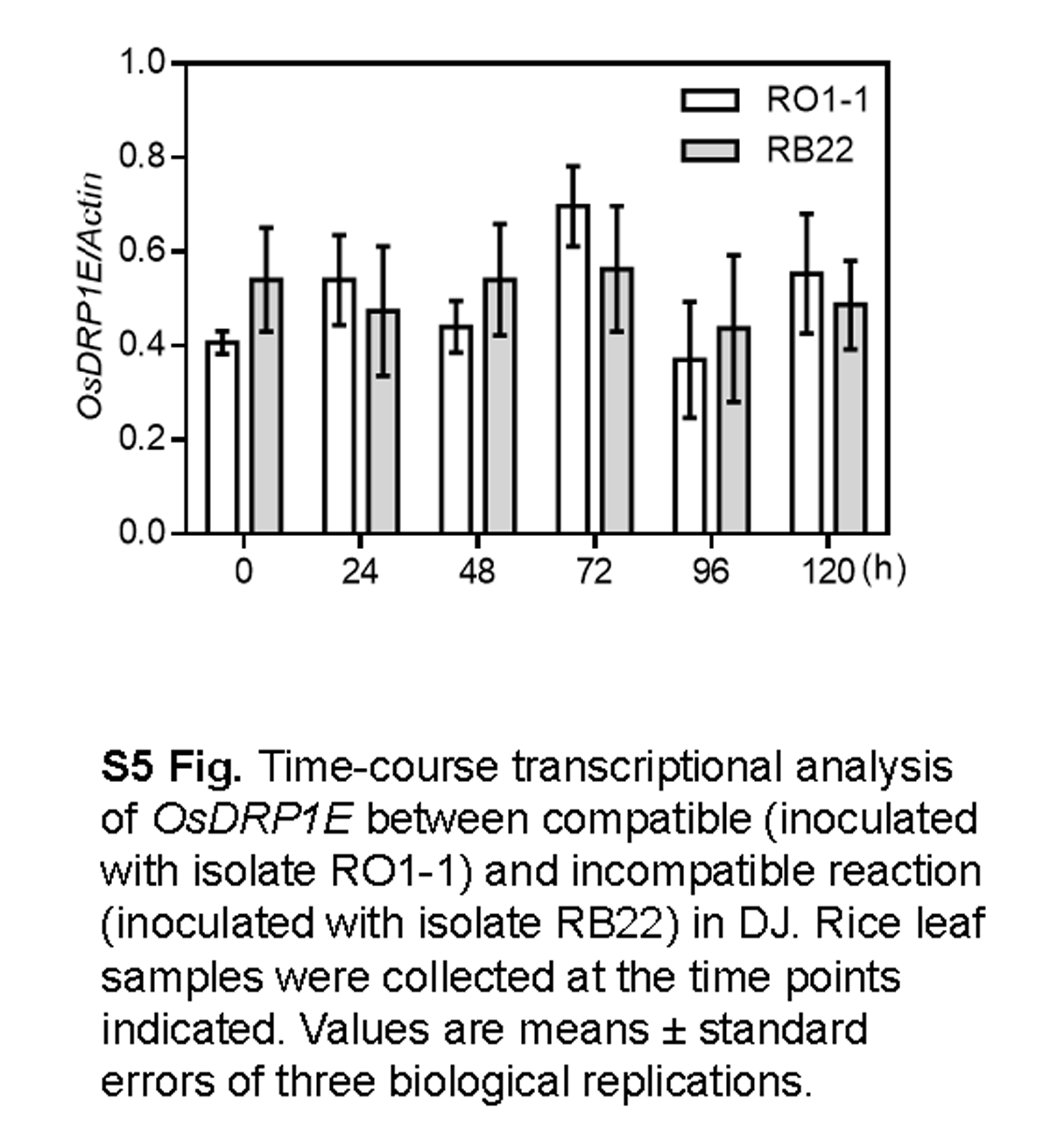

Supplement: S5 Fig — (TIF) [file ppat.1006157.s005.tif]

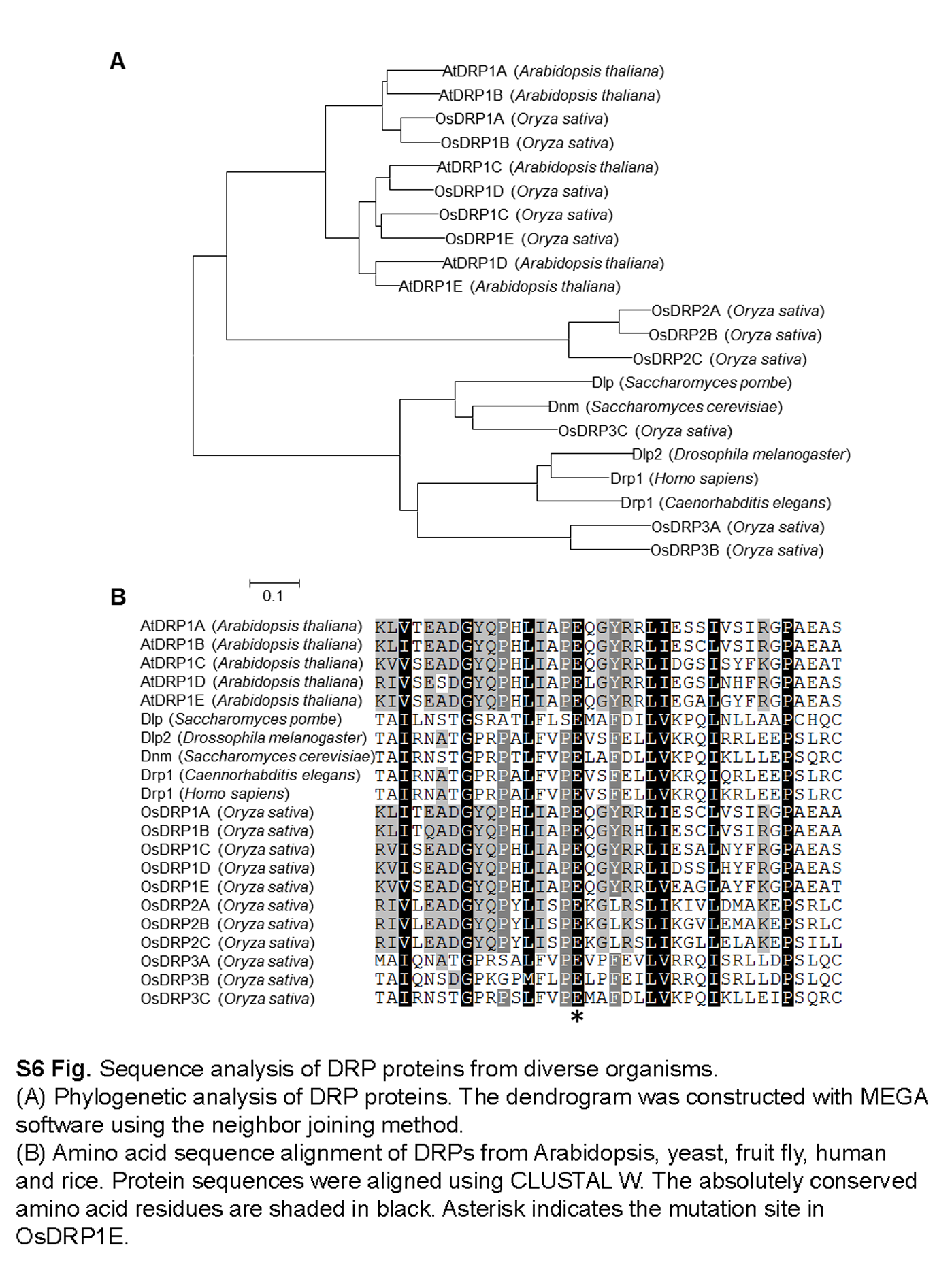

Supplement: S6 Fig — (TIF) [file ppat.1006157.s006.tif]

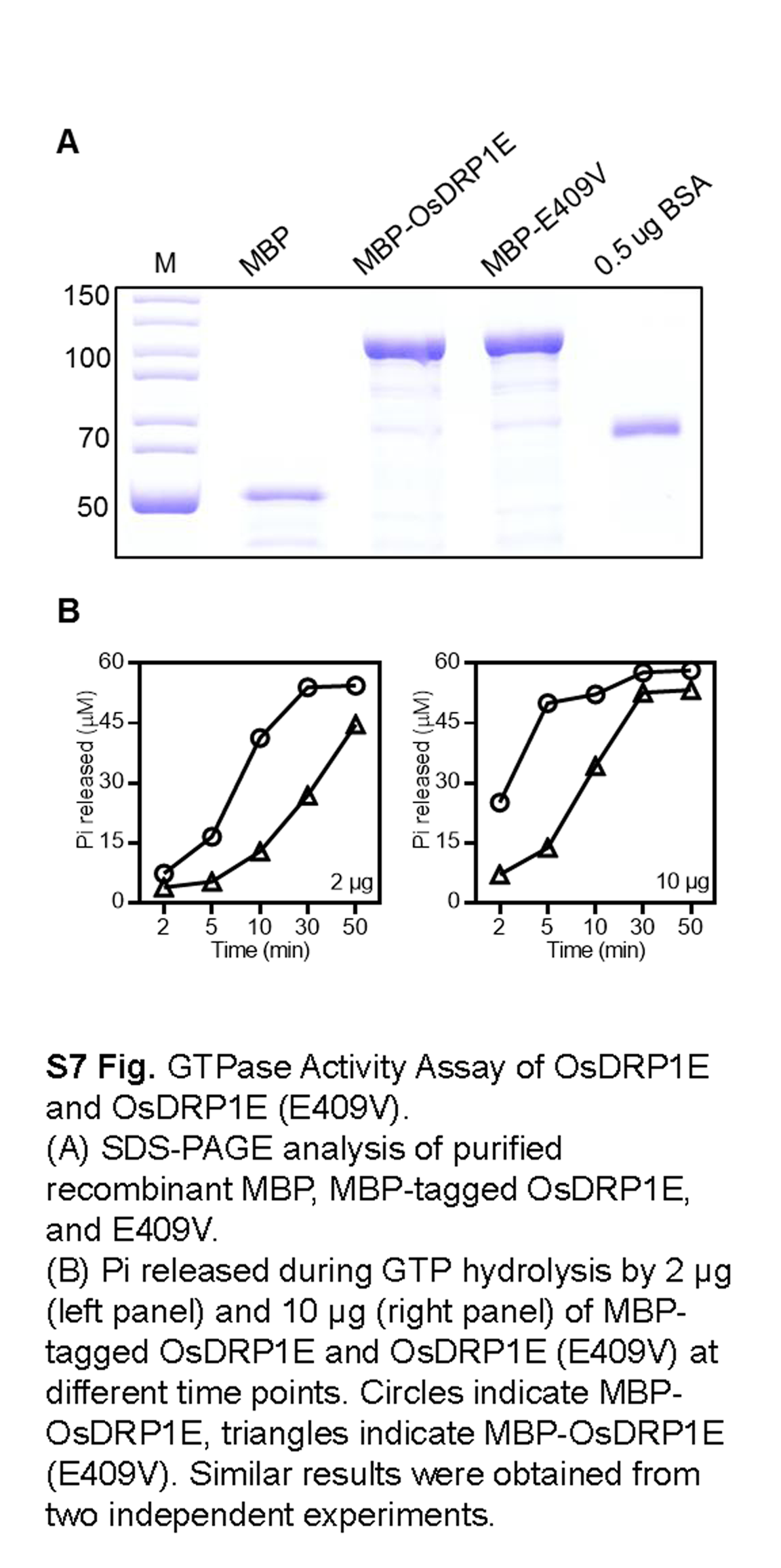

Supplement: S7 Fig — (TIF) [file ppat.1006157.s007.tif]

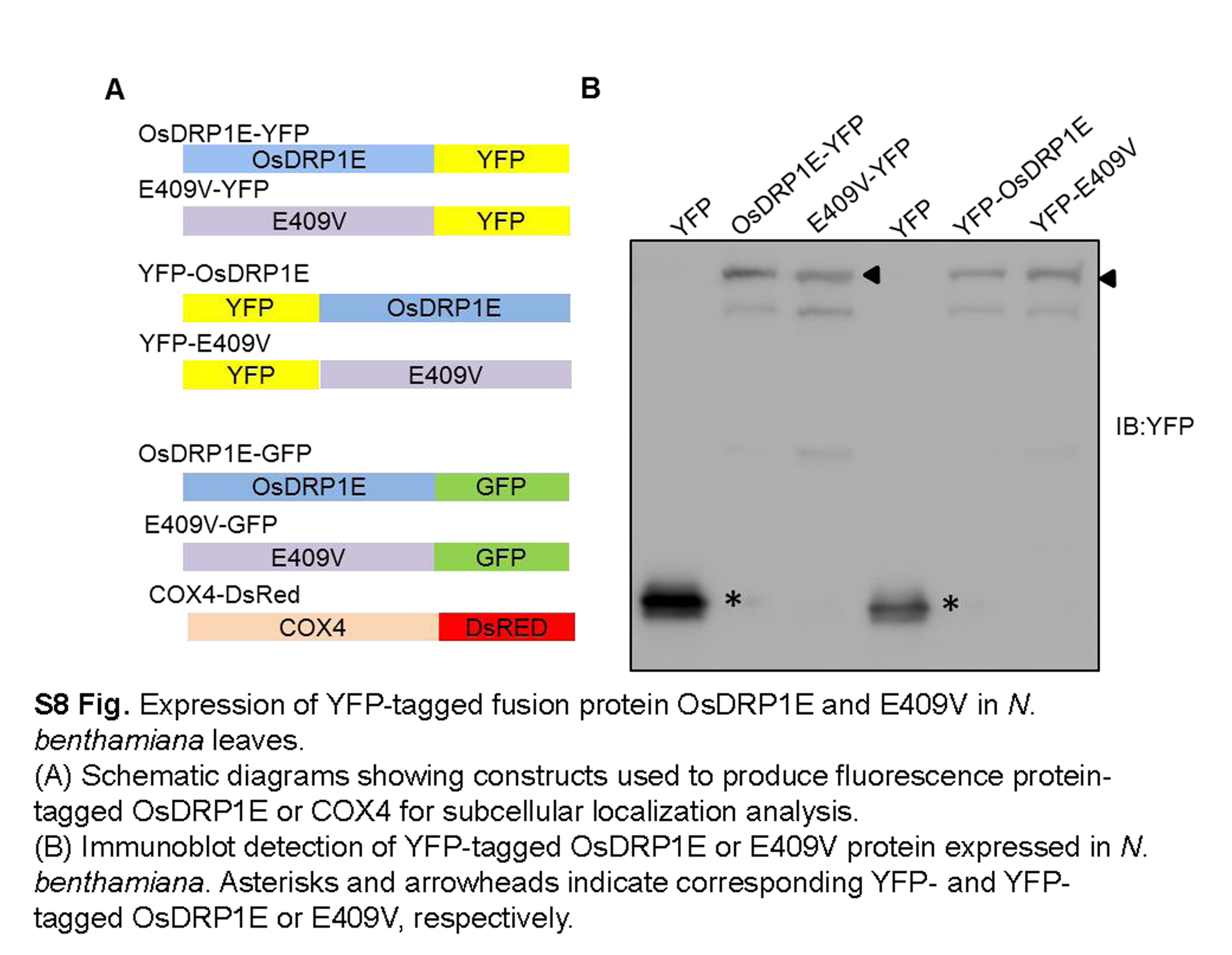

Supplement: S8 Fig — (TIF) [file ppat.1006157.s008.tif]

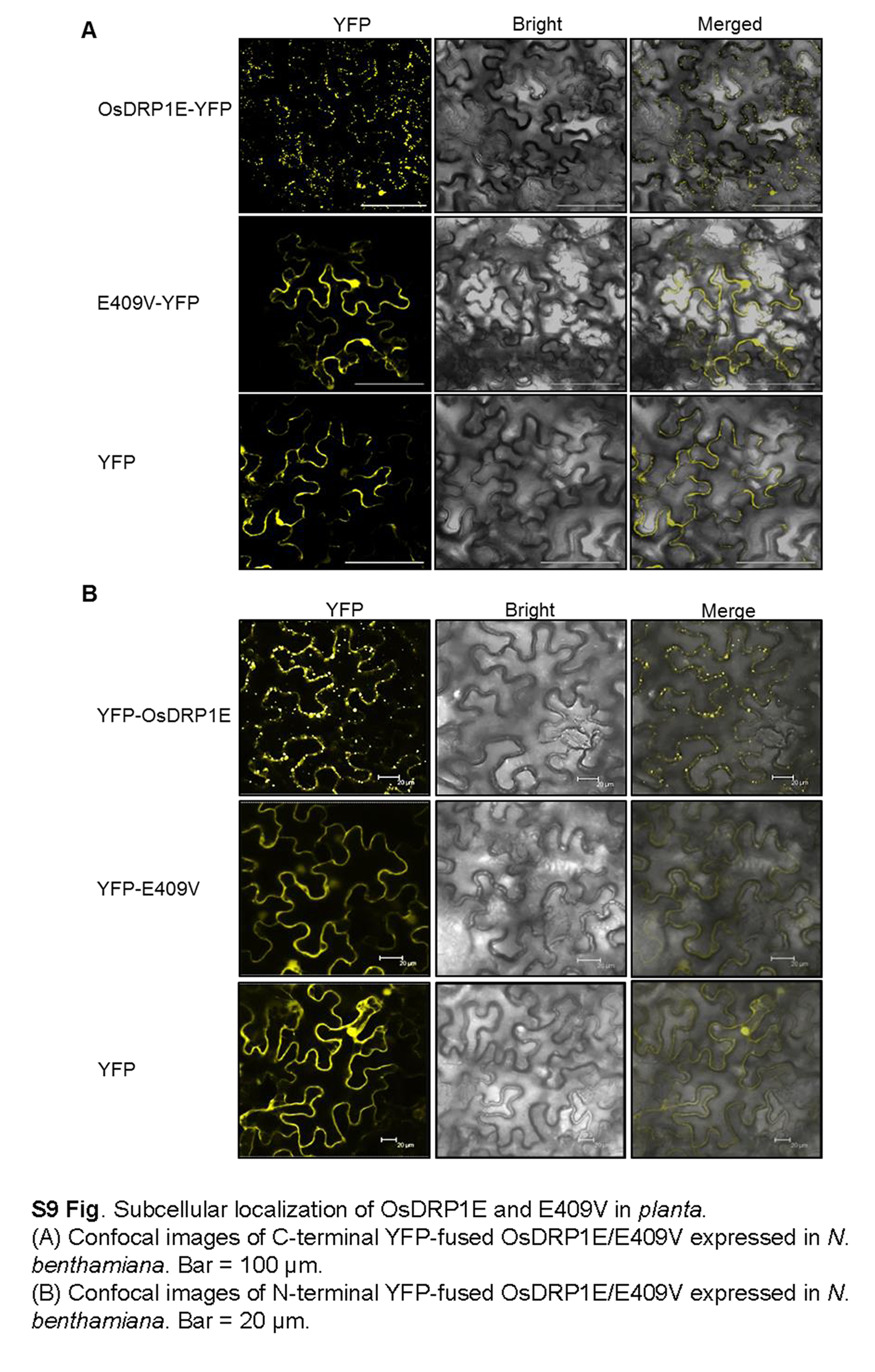

Supplement: S9 Fig — (TIF) [file ppat.1006157.s009.tif]

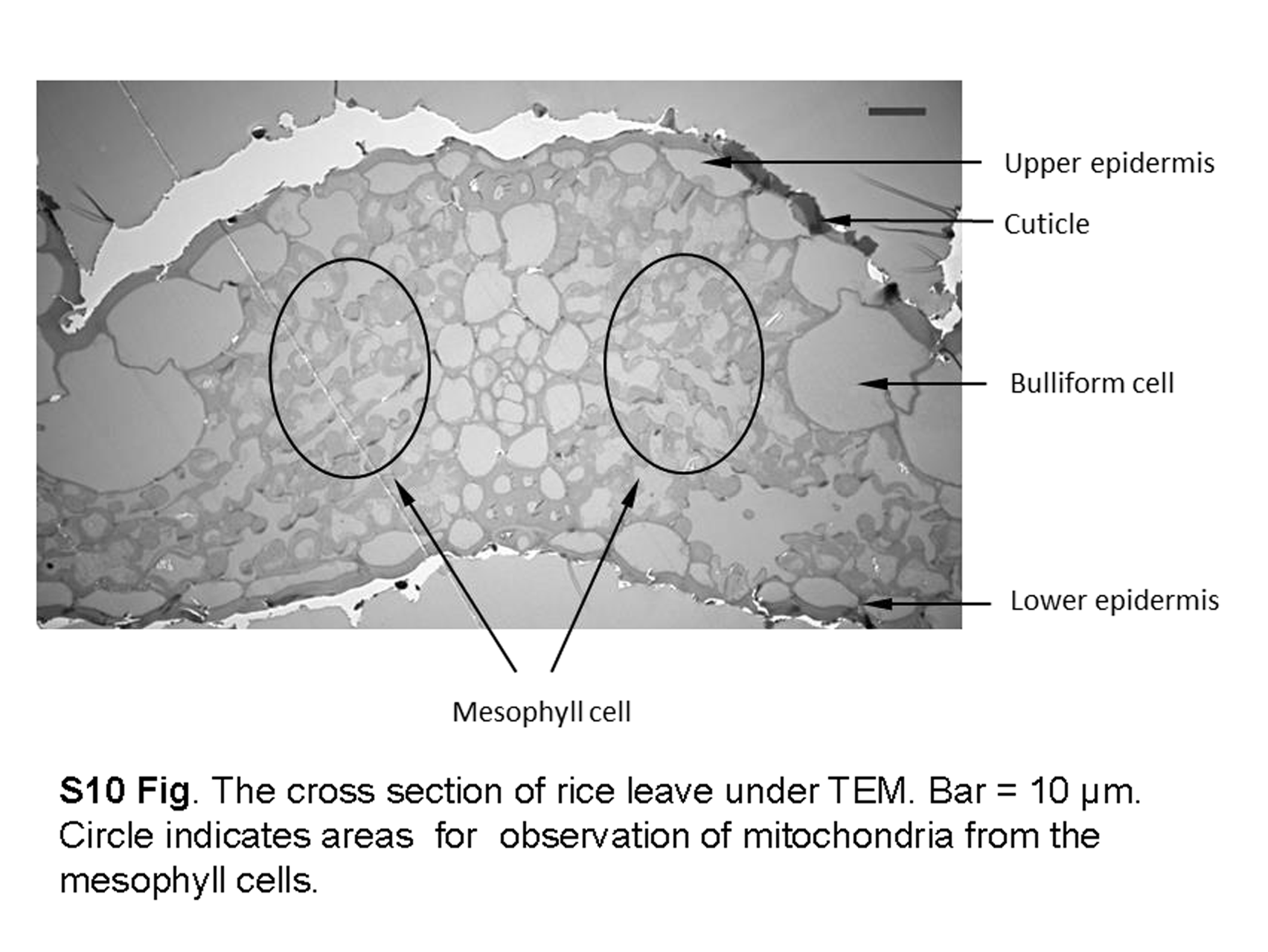

Supplement: S10 Fig — (TIF) [file ppat.1006157.s010.tif]

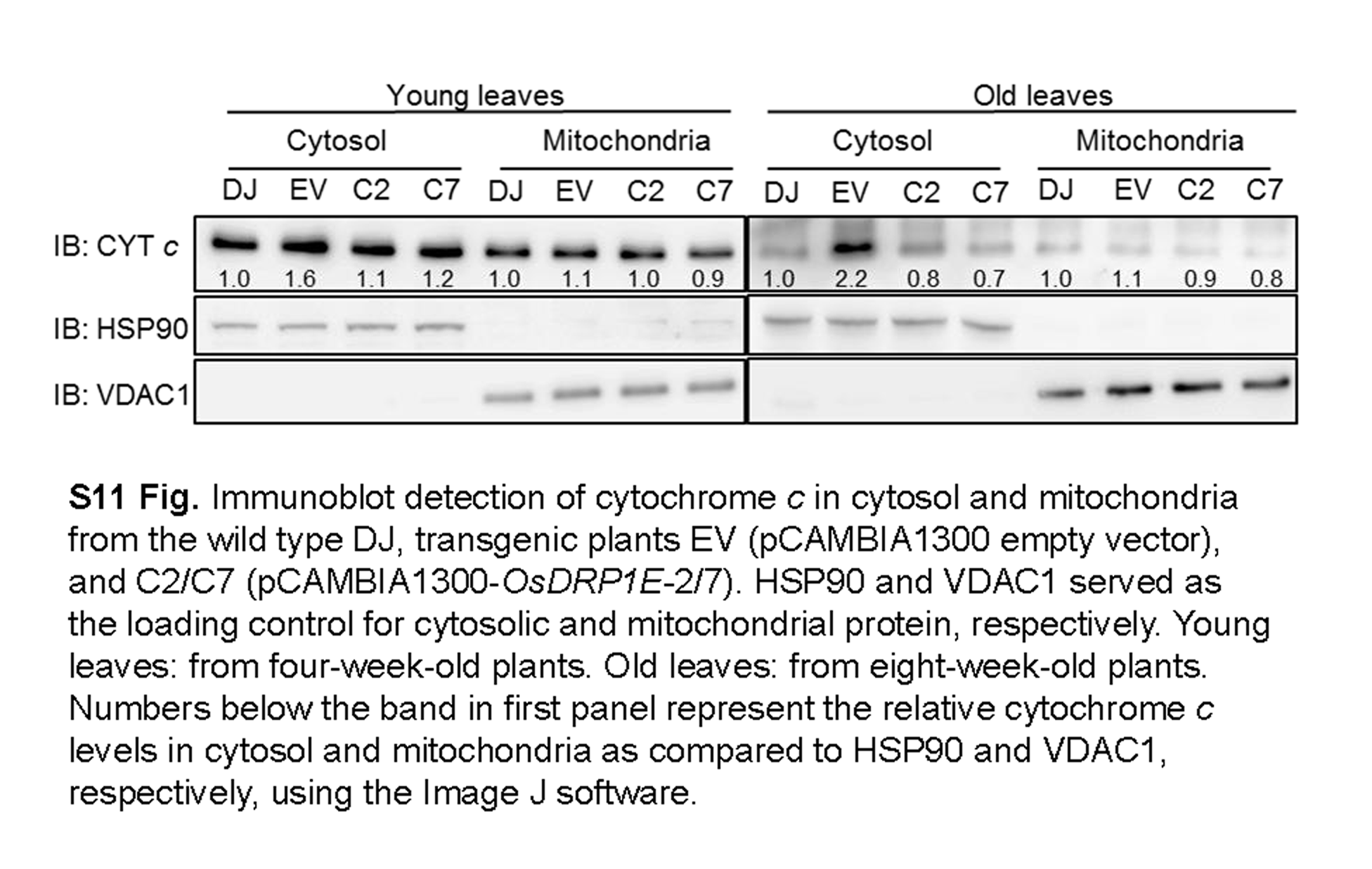

Supplement: S11 Fig — (TIF) [file ppat.1006157.s011.tif]
