# Supplementary material for: The Rice Dynamin-Related Protein OsDRP1E Negatively Regulates Programmed Cell Death by Controlling the Release of Cytochrome c from Mitochondria
Source: PLoS Pathog. 2017 Jan 12;13(1):e1006157. doi: 10.1371/journal.ppat.1006157 (PMC5266325; doi:10.1371/journal.ppat.1006157)
Supplement: S1 Table — (DOCX) [file ppat.1006157.s012.docx]

**S1 Table.** Agronomic traits of DJ and *dj-lm plants*

| Material | Plant height (cm) | Seed setting rate (%) | Tiller numbers | Flag leaf angle(°) | 1000-grain weight (g) | Panicle length (cm) |
| --- | --- | --- | --- | --- | --- | --- |
| DJ | 90.86±0.27 | 98.24±0.18 | 38±1 | 10.80±0.40 | 23.34±0.11 | 18.95±0.34 |
| *dj-lm* | 82.55±0.36*** | 61.12±1.78*** | 18±1*** | 51.25±2.34*** | 21.32±0.08*** | 17.83±0.31* |

Significance was determined at ***P<0.0001 or **P<0.01 or *P<0.05 with a Student’s *t*-test.
