# Supplementary material for: The Rice Dynamin-Related Protein OsDRP1E Negatively Regulates Programmed Cell Death by Controlling the Release of Cytochrome c from Mitochondria
Source: PLoS Pathog. 2017 Jan 12;13(1):e1006157. doi: 10.1371/journal.ppat.1006157 (PMC5266325; doi:10.1371/journal.ppat.1006157)
Supplement: S2 Table — (DOCX) [file ppat.1006157.s013.docx]

**S2 Table.** Genetic analysis of F_2_ populations

| Cross | Total number of plants | NO. of normal plants | NO. of lesion mimic plants | χ^2^(3:1) | χ^2^_0.05_ |
| --- | --- | --- | --- | --- | --- |
| 9311 × *dj-lm* | 126 | 87 | 39 | 2.4 | 3.84 |
| Dongjin × *dj-lm* | 133 | 96 | 37 | 0.49 | 3.84 |
