# Supplementary material for: The Rice Dynamin-Related Protein OsDRP1E Negatively Regulates Programmed Cell Death by Controlling the Release of Cytochrome c from Mitochondria
Source: PLoS Pathog. 2017 Jan 12;13(1):e1006157. doi: 10.1371/journal.ppat.1006157 (PMC5266325; doi:10.1371/journal.ppat.1006157)
Supplement: S3 Table — (DOCX) [file ppat.1006157.s014.docx]

**S3 Table.** Information about candidate genes

| Gene Locus. | Protein | Genomic sequence length | CDS length | Protein length |
| --- | --- | --- | --- | --- |
| LOC_Os09g39940.1 | plastocyanin-like domain containing protein, putative, expressed | 958 | 519 | 172 |
| LOC_Os09g39950.1 | POEI23 - Pollen Ole e I allergen and extensin family protein precursor, expressed | 711 | 504 | 167 |
| LOC_Os09g39960.1 | dynamin family protein, putative, expressed | 5278 | 1881 | 626 |
| LOC_Os09g39970.1 | expressed protein | 8389 | 3408 | 1135 |
| LOC_Os09g39980.1 | ubiquitin family protein, putative, expressed | 3274 | 669 | 222 |
| LOC_Os09g40000.1 | AGAP005770-PA, putative, expressed | 5208 | 1851 | 616 |
