# Supplementary material for: The Rice Dynamin-Related Protein OsDRP1E Negatively Regulates Programmed Cell Death by Controlling the Release of Cytochrome c from Mitochondria
Source: PLoS Pathog. 2017 Jan 12;13(1):e1006157. doi: 10.1371/journal.ppat.1006157 (PMC5266325; doi:10.1371/journal.ppat.1006157)
Supplement: S4 Table — (DOCX) [file ppat.1006157.s015.docx]

**S4 Table**. Primers used for fine mapping

| Name | Marker Type | F 5’-3’ | R 5’-3’ | Product Length |
| --- | --- | --- | --- | --- |
| RM24843 | SSR | GCGCGAATGCACGATTTAATTAGC | CTCATGACCGGGAAATCAACACG | 407 |
| ZQ-6 | Indel | AGTTCTCCACCATCACCGC | AACACCTCCCCAAATCCGA | 247 |
| ZQ-8 | Indel | CTATTCTTCTATTTCCCCC | AGTTCCTTTTCAAGCTTTT | 151 |
| ZQ-13 | Indel | AGACGATAGGGGTGTAGGT | GAATGAAGGCTTTTGTGAC | 317 |
| ZQ-14 | Indel | GGTTTACCGGCTTTCTATC | ACTCTCCCTCCACCATCTC | 205 |
| ZQ-18 | Indel | AGCCGCTCTTCTCGTCTCTT | TGGTGGTCGTCTTCCTCTTC | 268 |
| ZQ-26 | Indel | GCGAGTTTGAAAGTGAGCT | CATTAACGGCGAGTTGTCT | 238 |
