# Supplementary material for: The Rice Dynamin-Related Protein OsDRP1E Negatively Regulates Programmed Cell Death by Controlling the Release of Cytochrome c from Mitochondria
Source: PLoS Pathog. 2017 Jan 12;13(1):e1006157. doi: 10.1371/journal.ppat.1006157 (PMC5266325; doi:10.1371/journal.ppat.1006157)
Supplement: S5 Table — (DOCX) [file ppat.1006157.s016.docx]

**S5 Table.** Primers used for transcriptional analysis

| Name | F 5’-3’ | R 5’-3’ |
| --- | --- | --- |
| OsI85-qRT | GAGCAACGGCGTGGAGA | GCGGCGGTAGAGGAGATG |
| PR1a-qRT | GGAAGTACGGCGAGAACATC | TGGTCGTACCACTGCTTCTC |
| AOS2-qRT | CCCTAGCGTTGACAACAAGCA | CGGAGGTTGAAGCTTTGGTGA |
| PR5-qRT | TACAACGTCGCCATGAGCTTC | ACTTGGTAGTTGCTGTTGCCG |
| OsKS4-qRT | TCGCATTGCGTGTGCAA | TTGGAACTTCCGACATCGAAA |
| OsNAC4-qRT | TCCTGCCACCATTCTGAGATG | TTGCAGAATCATGCTTGCCAG |
| LOC_Os09g39940.1 | TCCAGGCGACCAGATAGTGT | GGGAAGCCACAGATGAAGTAG |
| LOC_Os09g39950.1 | TGGAGGGCGACGTGTACTGC | GTGGGCGGGTCTTGAGGAA |
| LOC_Os09g39960.1/  OsDRP1E | GGTTCGGTCGTATGTTGAGA | AGAGCGTTTGTTCCCTTGTC |
| LOC_Os09g39970.1 | CTTGGGAGCAGAAGATAGTAGA | AAATAGTCGGTGTTAAATAGCC |
| LOC_Os09g39980.1 | GCCTCCCTCCGTTTATCTC | CAGGTGGTTGTTTCTGGTATT |
| LOC_Os09g40000.1 | CCGATGAATACGGCAAGGT | GGCGTGGTAGAAGACGAAGG |
| Actin-qRT | TGAAGATCAAGGTGGTGGCAC | TGCTGGACCCGACTCATCATA |
