# Supplementary material for: The Rice Dynamin-Related Protein OsDRP1E Negatively Regulates Programmed Cell Death by Controlling the Release of Cytochrome c from Mitochondria
Source: PLoS Pathog. 2017 Jan 12;13(1):e1006157. doi: 10.1371/journal.ppat.1006157 (PMC5266325; doi:10.1371/journal.ppat.1006157)
Supplement: S6 Table — (DOCX) [file ppat.1006157.s017.docx]

**S6 Table.** Primers used for functional analysis of *OsDRP1E*.

| Name | 5’-3’ |
| --- | --- |
| Primer 1-F | TCCCCCGGGAATGGCGAGCATGGAGGGTCT |
| Primer 1-R | GGGGTACCCCTGGTCCATGCGACAGA |
| Primer 2-R | GACTAGTCTACCTGGTCCATGCGACAGA |
| Primer 3-F | GGGATCCATGGCGAGCATGGAGGGTCT |
| Primer 3-R | ACGCGTCGACCTACCTGGTCCATGCGACAGA |
| BQ5-2R-kpnI | GGGGTACCCACCCATAGCAGACAAAA |
| BQ4-1F | AGATTACCAGGACAGTTAGCA |
| BQ4-3F | CTGCGGAGAACTCATACAAATA |
| BQ3-1F-SalI | ACGCGTCGACTAATTGGGCCGTCGTTCCAT |
